# A single mutation in the E2 glycoprotein of hepatitis C virus broadens the claudin specificity for its infection

**DOI:** 10.1038/s41598-022-23824-3

**Published:** 2022-11-24

**Authors:** Yoshitaka Shirasago, Hidesuke Fukazawa, Shotaro Nagase, Yoshimi Shimizu, Tomoharu Mizukami, Takaji Wakita, Tetsuro Suzuki, Hideki Tani, Masuo Kondoh, Takuya Kuroda, Satoshi Yasuda, Yoji Sato, Kentaro Hanada, Masayoshi Fukasawa

**Affiliations:** 1 Department of Biochemistry and Cell Biology, National Institute of Infectious Diseases, 1-23-1 Toyama, Shinjuku-ku, Tokyo, 162-8640 Japan; 2 Department of Quality Assurance, Radiation Safety, and Information System, National Institute of Infectious Diseases, 1-23-1 Toyama, Shinjuku-ku, Tokyo, 162-8640 Japan; 3 Graduate School of Pharmaceutical Sciences, Osaka University, 1-6 Yamadaoka, Suita, Osaka 565-0871 Japan; 4 Department of Pharmaceutical Sciences, Teikyo Heisei University, 4-21-2 Nakano, Nakano-ku, Tokyo, 164-8530 Japan; 5 National Institute of Infectious Diseases, 1-23-1 Toyama, Shinjuku-ku, Tokyo, 162-8640 Japan; 6 Department of Infectious Diseases, Hamamatsu University School of Medicine, 1-20-1 Handayama, Higashi-ku, Hamamatsu, Shizuoka 431-3192 Japan; 7 Department of Virology, Toyama Institute of Health, 17-1 Nakataikouyama, Imizu, Toyama 939-0363 Japan; 8 Division of Cell-Based Therapeutic Products, National Institute of Health Sciences, 3-25-26 Tonomachi, Kawasaki-ku, Kawasaki, Kanagawa 210-9501 Japan

**Keywords:** Microbiology, Virology

## Abstract

Entry of the hepatitis C virus (HCV) into host cells is a multistep process mediated by several host factors, including a tight junction protein claudin-1 (CLDN1). We repeatedly passaged HCV-JFH1-tau, an HCV substrain with higher infectivity, on Huh7.5.1-8 cells. A multi-passaged HCV-JFH1-tau lot was infectious to CLDN1-defective S7-A cells, non-permissive to original HCV-JFH1-tau infection. We identified a single mutation, M706L, in the E2 glycoprotein of the HCV-JFH1-tau lot as an essential mutation for infectivity to S7-A cells. The pseudovirus JFH1/M706L mutant could not infect human embryonic kidney 293 T (HEK293T) cells lacking CLDN family but infected HEK293T cells expressing CLDN1, CLDN6, or CLDN9. Thus, this mutant virus could utilize CLDN1, and other CLDN6 and CLDN9, making HCV possible to infect cells other than hepatocytes. iPS cells, one of the stem cells, do not express CLDN1 but express CLDN6 and other host factors required for HCV infection. We confirmed that the HCV-JFH1-tau-derived mutant with an M706L mutation infected iPS cells in a CLDN6-dependent manner. These results demonstrated that a missense mutation in E2 could broaden the CLDN member specificity for HCV infection. HCV may change its receptor requirement through a single amino acid mutation and infect non-hepatic cells.

## Introduction

The hepatitis C virus (HCV), first identified in 1989, is an enveloped positive-stranded RNA virus belonging to the *Flaviviridae* family. HCV remains a major global health problem, with approximately 71 million people currently infected worldwide^1^. These people have chronic HCV infection, putting these individuals at high risk for progressive liver disease, including cirrhosis, hepatocellular carcinoma, and other extra-hepatic lesions^2^.

The HCV genome includes over 9,400 nucleotides. Its genome heterogeneity, namely quasispecies diversity, is one of the main features of HCV. The underlying reason for such variability is a lack of the corrective activity of virus-dependent RNA-polymerase leading to the frequent introduction of nucleotide substitutions in the virus genome^3^.

The entry of HCV into cells is a complex, multistep process requiring the two viral envelope glycoproteins, E1 and E2, as well as various host factors, such as the scavenger receptor class B type I (SRBI)^4^, the cholesterol transporter Niemann-Pick disease type C1 like 1^5^, epidermal growth factor receptor^6^, the cluster of differentiation 81 (CD81) molecule^7^, claudin-1 (CLDN1)^8,9^, and occludin (OCLN)^10–13^. These host factors are believed to be involved in HCV entry co-operatively, and sequential post binding usage of SRBI, CD81, CLDN1, and OCLN^14^ are at least predicted. However, the molecular mechanism remains poorly understood^15^.

This study isolated and characterized an HCV substrain infectious to CLDN1-defective cells and found that this substrain attained alternative CLDNs selectivity only by a single amino acid mutation in the E2 glycoprotein of HCV-JFH1-tau. Furthermore, we also demonstrated that this substrain could infect non-hepatic iPS cells, suggesting a possible relationship between HCV glycoprotein mutations and extra-hepatic infection.

## Results

### Isolation of HCV-JFH1-tau substrains permissive to CLDN1-defective S7-A cells

**Figure 1:**
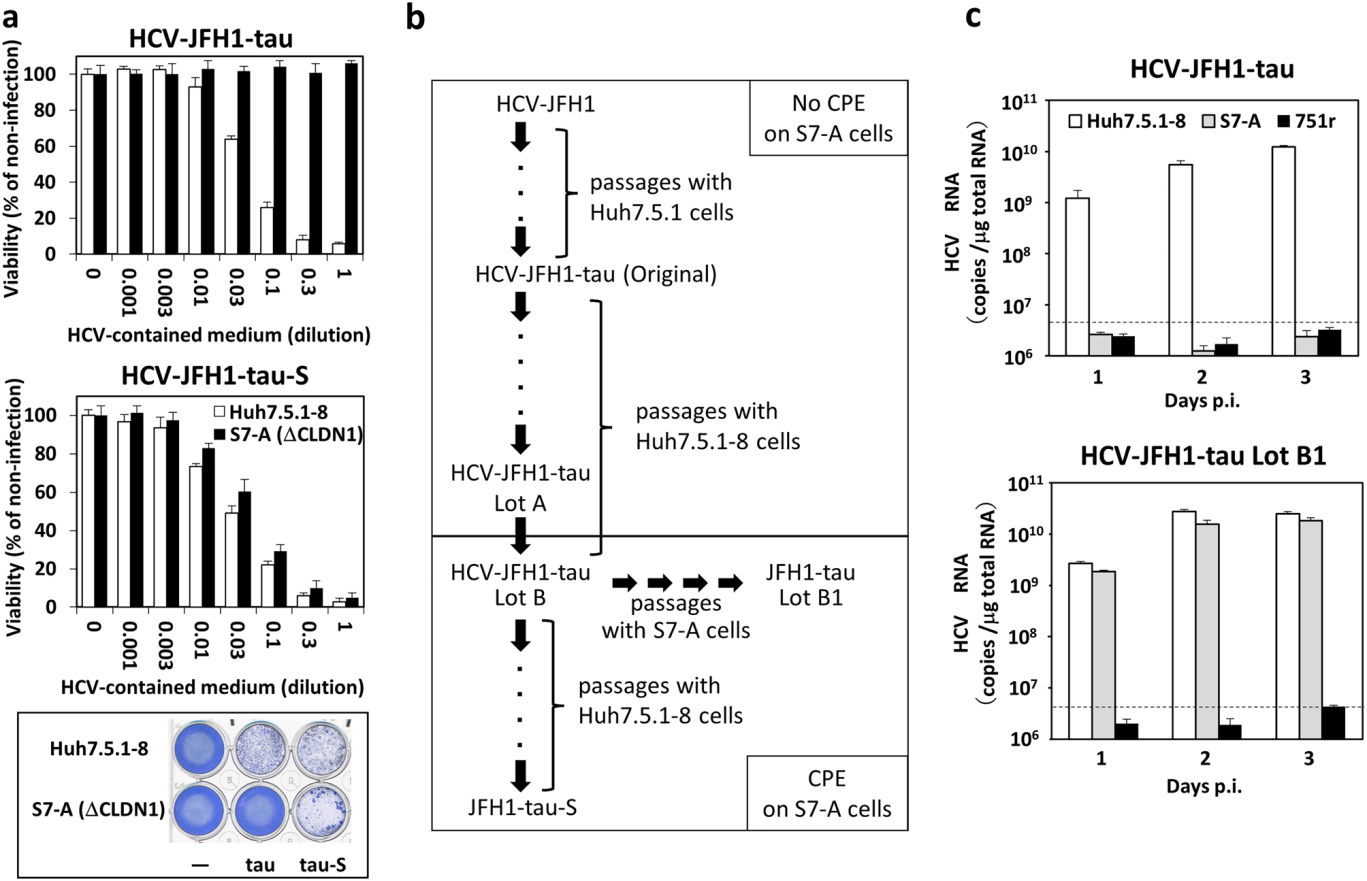
Characterization of HCV-JFH1-tau substrains. (**a**) The CPE of HCV-JFH1-tau-S on CLDN1-defective S7-A cells. Huh7.5.1-8 and S7-A cells were infected with HCV-JFH1-tau at 1.7 × 10^6^ Geq/cell or HCV-JFH1-tau-S at 7.2 × 10^5^ Geq/cell. Four days postinfection (p.i.), cell viability was measured by MTT assay. White bars, Huh7.5.1-8 cells; black bars, S7-A cells. Values of viability in each cell are expressed as percentages of values from each naive cell. Data are presented as means ± SD (n = 3). Coomassie Brilliant Blue stain images of Huh7.5.1-8 or S7-A cells treated (HCV-JFH1-tau, HCV-JFH1-tau-S) or not treated (−) with no dilution of HCV-contained medium were also shown (bottom), (**b**) Flow chart for the isolation of HCV-JFH1-tau-S and HCV-JFH1-tau Lot B1. (**c**) Huh7.5.1-8, S7-A, or 751r cells were infected with HCV-JFH1-tau or HCV-JFH1-tau Lot B1 at 1.0 × 10^4^ Geq/cell. 1–3 days p.i., cellular HCV RNA was measured by qRT-PCR (n = 4). Dashed lines in **c** are the limit of detection, whose values were (1.50 ± 0.27) × 10^6^ copies /μg total RNA, calculated from the values of no infection.

Previously, we demonstrated that the HCV-JFH1-tau substrain having K74T/I414T mutation has higher infectivity and shows cytopathic effects (CPE) on Huh7.5.1-8 cells, compared to the wild-type HCV-JFH1^16^. We repeatedly passaged HCV-JFH1-tau substrains with Huh7.5.1-8 cells and stocked them at each passage. Surprisingly, we found that a multi-passaged HCV-JFH1-tau lot, designated as HCV-JFH1-tau-S, showed CPE on CLDN1-defective S7-A cells, non-permissive to original HCV-JFH1 and HCV-JFH1-tau infection^9,16^. Cell viability using a tetrazolium-based colorimetric assay (MTT assay) was performed to confirm this effect (Fig. 1a). When Huh7.5.1-8 cells and S7-A cells were infected with original (parental) HCV-JFH1-tau, the viability of Huh7.5.1-8 cells was decreased in the viral dose-dependent manner; however, the viability of S7-A cells was not affected even at the maximum viral load. Conversely, when Huh7.5.1-8 cells and S7-A cells were infected with HCV-JFH1-tau-S, the viability of Huh7.5.1-8 cells and S7-A cells was similarly decreased in a viral dose-dependent manner. Then we checked the CPE of all passaged viral stocks on S7-A cells and got the first lot showing CPE on S7-A cells, designated as HCV-JFH1-tau Lot B. The lot just before HCV-JFH1-tau Lot B was named HCV-JFH1-tau Lot A (Fig. 1b), showing no CPE on S7-A cells. Since there was a possibility that HCV-JFH1-tau Lot B consists of various substrains containing original HCV-JFH1-tau, HCV-JFH1-tau Lot B was passaged four times with S7-A cells to purify a population permissive to S7-A cells. The passaged lot was named HCV-JFH1-tau Lot B1 (Fig. 1b). We then examined whether HCV-JFH1-tau Lot B1 is infectious to S7-A cells (Fig. 1C and Supplementary Fig. S1). Huh7.5.1-8, S7-A, and CD81-defective 751r cells were incubated with parental HCV-JFH1-tau or HCV-JFH1-tau Lot B1, and then HCV RNA contents in the cells (Fig. 1c) and supernatants (Supplementary Figs. S1a and S1b) were determined 1–3 days postinfection (p.i.). Huh7.5.1-8 cells were permissive to both HCV-JFH1-tau and HCV-JFH1-tau Lot B1 infection, and 751r cells were non-permissive to both virus infections, suggesting that HCV-JFH1 Lot B1 infection depends on CD81. In contrast, S7-A cells were non-permissive to HCV-JFH1-tau infection but permissive to HCV-JFH1-tau Lot B1 infection. Consistent with these results, immunoblot analyses using antibodies against HCV core and NS3 proteins (Supplementary Figs. S1c and S1d) and immunofluorescent microscopic analyses using an antibody against HCV core protein (Supplementary Figs. S1e and S1f.) showed that HCV-JFH1-tau Lot B1 could infect CLDN1-defective S7-A cells. We also determined viral titers of HCV-JFH1-tau Lot B1 using Huh7.5.1-8 and S7-A cells. The viral titer of HCV-JFH1-tau Lot B1 for S7-A cells was similar to those of HCV-JFH1-tau Lot B1 and parental HCV-JFH1-tau for Huh7.5.1-8 cells (Supplementary Table S1). These results indicated that HCV-JFH1-tau Lot B1 could infect cells without using CLDN1. We further verified the dependence of HCV-JFH1-tau Lot B1 infection on CLDN1 using previously developed anti-CLDN1 monoclonal antibodies (mAbs)^9^. When Huh7.5.1-8 cells were infected with HCV-JFH1-tau or HCV-JFH1-tau Lot B1 in the presence of anti-CLDN1 mAbs (clone 2C1 or 3A2), genomic RNA and protein productions of HCV-JFH1-tau were severely blocked by anti-CLDN1 mAbs, but those of HCV-JFH1-tau Lot B1 were not (Supplementary Fig. S2). From these results, we confirmed that HCV-JFH1-tau Lot B1 could infect cells, not via CLDN1.

We next evaluated the dependence of HCV-JFH1-tau Lot B1 on OCLN, another tight junction protein reported to be essential for infection with various HCV genotypes^10–12^. Huh7.5.1-8, S7-A, and OKH-4 cells, Huh7.5.1-8-derived OCLN-knockout cells^11^, were incubated with HCV-JFH1-tau or HCV-JFH1-tau Lot B1, and then cellular HCV RNA contents 4 days p.i. were determined by qRT-PCR. As previously reported^16^, HCV-JFH1-tau RNA was only detected in Huh7.5.1-8 cells. HCV-JFH1-tau Lot B1 RNA was detected in both Huh7.5.1-8 and S7-A cells but not in OKH-4 cells (Supplementary Fig. S3a). Data from immunoblot analyses of cellular HCV core and NS3 proteins were consistent with these results (Supplementary Fig. S3b). These results indicated that HCV-JFH1-tau Lot B1 infection as well as HCV-JFH1-tau infection depend on OCLN.

### Determination of envelope amino acid residues in HCV-JFH1-tau Lot B1 involved in its infection of CLDN1-defective S7-A cells

Owing to the viral genomic mutations, HCV-JFH1-tau Lot B1 should acquire the CLDN1-independent infection (entry) phenotype. Thus, we first determined the nucleotide sequences for the envelope protein region of HCV-JFH1-tau Lot B1 and HCV-JFH1-tau Lot A, which is involved in viral entry steps. HCV-JFH1-tau, showing higher infectivity, has two amino acid mutations (K74T in the core protein and I414T in the E2 protein)^16^, compared to the original clinical isolate HCV-JFH1. HCV-JFH1-tau Lot A, exhibiting the same phenotype as parental HCV-JFH1-tau, had four additional nonsynonymous amino acid mutations: N234D and V293A in the E1 protein, as well as V402A and Y443H in the E2 protein (Table 1). HCV-JFH1-tau Lot B1 had additional eleven nonsynonymous amino acid mutations: A218G, T260A, Q296R, H316N, T331S, A351D, and I374V in the E1 protein, and V392A, A464D, M706L, and V724I in the E2 protein compared to HCV-JFH1-tau Lot A (Table 1).

**Table 1:** Mutation sites in the envelop proteins of HCV-JFH1 substrains.

No	Protein	Position of amino acids	HCV-JFH1			
			Original	HCV-JFH1-tau		
				Original	Lot A	Lot B1
1	E1	218	A(GCT)			G(GGT)
2		234	N(AAT)		D(GAC)	
3		260	T(ACG)			A(GCG)
4		293	V(GTC)		A(GCC)	
5		296	Q(CAG)			R(CGG)
6		316	H(CAC)			N(AAC)
7		331	T(ACC)			S(TCC)
8		351	A(GCT)			D(GAT)
9		374	I(ATC)			V(GTC)
10	E2	392	V(GTT)			A(GCT)
11		402	V(GTG)		A(GCG)	
12		414	I(ATT)	T(ACT)		
13		443	Y(TAC)		H(CAC)	
14		464	A(GCT)			D(GAT)
15		706	M(ATG)			L(TTG)
16		724	V(GTA)			I(ATA)

Then we tried to identify the residues among eleven mutated amino acids in HCV-JFH1-tau Lot B1 responsible for the CLDN1-independent infection phenotype using an infection system where infection assays are performed by HCV-JFH1-tau encapsidated with exogenously supplemented HCV envelope proteins with mutations (HCVee) (Supplementary Fig. S4a).

We first evaluated whether the HCVee infection system functions well in our experimental conditions. Huh7.5.1-8 cells were infected with HCV-JFH1-tau at a multiplicity of infection (MOI) of 1 and cultured for 2 days. And then, infected cells were transfected with plasmids encoding envelope proteins from HCV-JFH1 or HCV-JFH1-tau Lot B1. After 3 days, the supernatant that may contain HCVee, produced from these infected cells, were collected and infected naive Huh7.5.1-8, S7-A, and 751r cells. HCVee infectivity was determined by immunohistochemistry using anti-HCV core protein mAb. HCV particles in the culture supernatant from the infected cells expressing HCV-JFH1-tau envelope did not infect S7-A cells. However, those in the culture supernatant from the infected cells expressing HCV-JFH1-tau Lot B1 envelope infected S7-A cells and Huh7.5.1-8 cells (Supplementary Fig. S4b). These results show that HCVee infection system is working well.

Next, to identify mutation sites involved in the CLDN1-independent infection by HCV-JFH1-tau Lot B1, we tested the infectivity of HCVee having Lot B1-type envelope proteins with each wild-type point mutation. When these eleven kinds of HCVee inoculated S7-A cells, Lot B1-based HCVee carrying wild-type point mutation: A218, T260, Q296, H316, T331, A351, T374, A464, or V724 could infect S7-A cells, but those carrying V392 or M706 reverting mutation were not (Supplementary Fig. S5 and Table 2). Furthermore, all HCVee tested were permissive to Huh7.5.1-8 cells but not to 751r cells (Supplementary Fig. S5). These results suggested that V392A and M706L mutations in HCV-JFH1-tau Lot B1 were involved in its CLDN1-independent infection.

**Table 2:** Amino acid residues in the envelope proteins of HCV-JFH1-tau Lot B1　involved in its infection of S7-A cells.

Each wild-type point mutation in HCV-JFH1-tau Lot B1-based HCVee	Infectivity to S7-A cells
A218	+
T260	+
Q296	+
H316	+
T331	+
A351	+
T374	+
V392	−
A464	+
M706	−
V724	+

### Introduction of an M706L point mutation into HCV-JFH1 leads to the acquisition of CLDN1-independent infection phenotype

**Figure 2:**
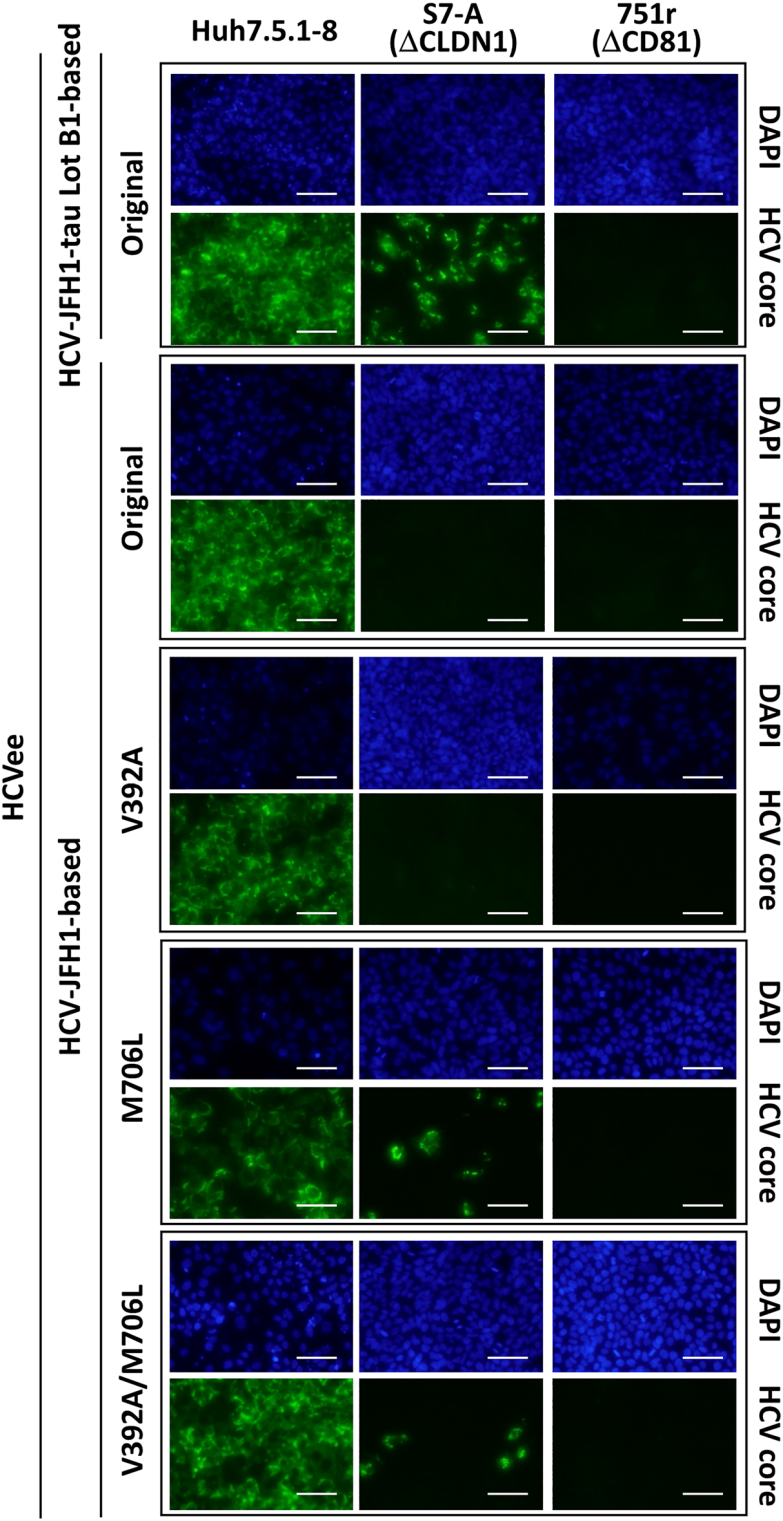
M706L mutation in HCVee is required for its infection of CLDN1-defective S7-A cells. HCVee preparation and infection were performed as described in Materials and Methods, in which pcDNA3.1( +)-HCV-ΔC-E2 plasmids based on HCV-JFH1-tau Lot B1-type plasmid or wild-type plasmids with or without V392A, M706L, or V392A/M706L mutation were used. HCVee-infected Huh7.5.1-8, S7-A, and 751r cells were stained with anti-HCV core protein mAb (green) and DAPI (blue) and observed using fluorescence microscopy. Bars, 50 μm.

**Figure 3:**
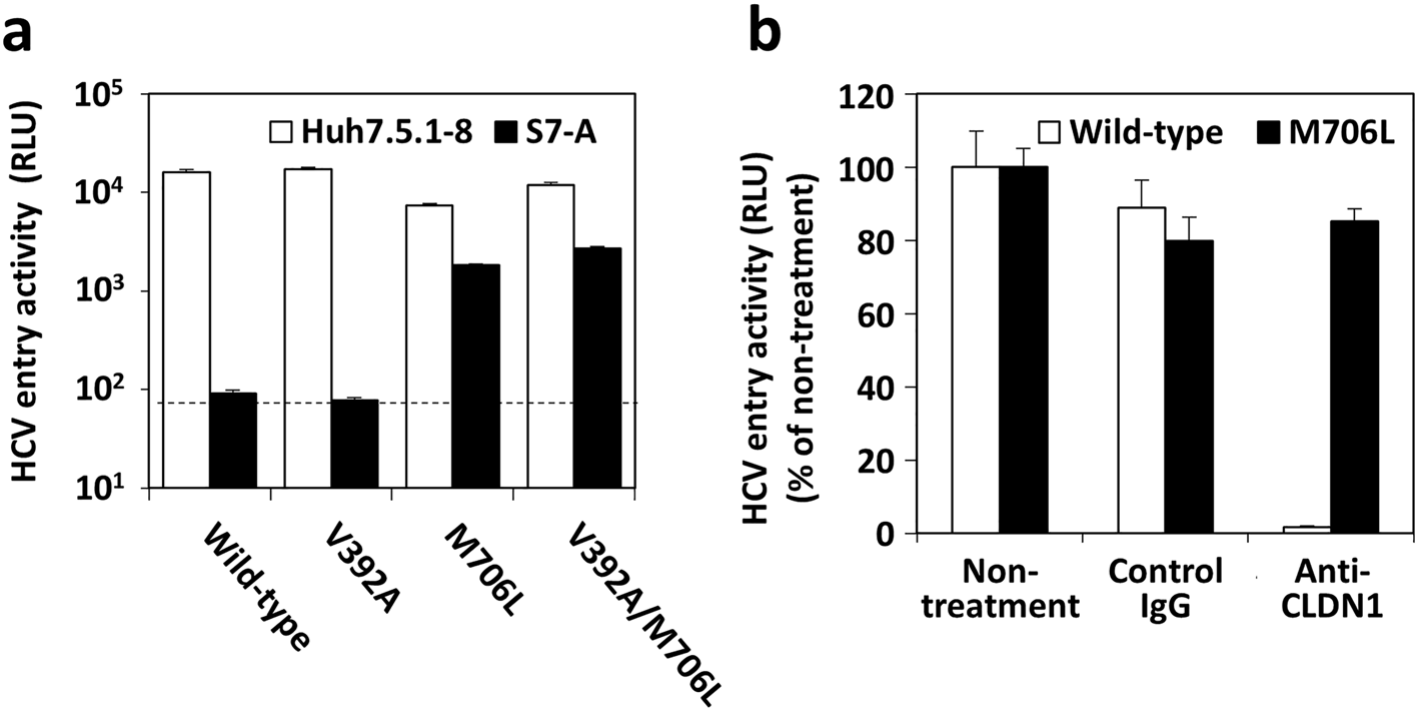
HCVpp carrying M706L mutation can infect cells via CLDN1-independent pathway. (**a**) Huh7.5.1-8 (white) and S7-A (black) cells were infected with HCV-JFH1-based HCVpp (Wild-type, V392A, M706L, and V392A/M706L) for 6 h. Two days p.i., luciferase activities (relative luminescence units: RLU) of cell lysates were measured using a luminometer. Data are presented as the mean ± S.D. (n = 3). The dashed line in A is the detection limit, whose value was (8.7 ± 0.5) × 10^1^. (**b**) Huh7.5.1-8 cells were preincubated with 20 μg/ml of each control mouse IgG or anti-CLDN1 mAb (2C1) for 30 min at room temperature and then infected with HCVpp (Wild-type or M706L) for 6 h. Two days p.i., luciferase activities of cell lysates were measured using a luminometer. In (**b**), values of luciferase activities were expressed as percentages of control values (treatment without antibodies). Data are presented as the mean ± S.D. (n = 3).

Conversely, we then tested whether introducing V392A and/or M706L mutations into HCV-JFH1 leads to the acquisition of infectivity to S7-A cells. HCV-JFH1-based HCVee with M706L or V392A/M706L mutation and Lot B1-based HCVee can infect S7-A cells, but HCV-JFH1-based HCVee with or without V392A mutation cannot (Fig. 2). These results revealed that the CLDN1-independent infection phenotype could be acquired by only M706L mutation in HCV-JFH1. To confirm these, we further examined the characteristics of V392A and M706L mutant viruses using the HCV pseudoparticle (HCVpp) system. HCV-JFH1-based HCVpps with M706L or V392A/M706L mutation could infect S7-A cells, whereas those with no mutation (wild-type) or V392A mutation could not; although all HCVpps tested can infect Huh7.5.1-8 cells (Fig. 3a). Moreover, infection of Huh7.5.1-8 cells with wild-type HCV-JFH1-based HCVpp was strongly inhibited by anti-CLDN1 mAb, whereas infection of Huh7.5.1-8 cells with HCV-JFH1-based HCVpp having M706L was not (Fig. 3b). These results using HCVpps were consistent with those using the HCVee infection system. Altogether we concluded that M706L mutation in HCV-JFH1 is essential for its CLDN1-independent infection phenotype.

Additionally, infection with HCV-JFH1-based HCVpp having M706L mutation, similar to that with wild-type HCV-JFH1-based HCVpp, showed a CD81-, SRBI-, and OCLN-dependent manner (Supplementary Fig. S6).

### M706L mutation in HCV-JFH1 expands entry receptor dependency from CLDN1 to other CLDN family proteins: CLDN6 and CLDN9

What receptors, other than CLDN1, does HCV-JFH1-based HCVpp having M706L mutation use in the entry step? We had a hint through our trial experiments using M706L-carrying HCV-JFH1-tau Lot B1 and hepatic CLDN1-defective S7-A cells. When S7-A cells were infected with HCV-JFH1-tau Lot B1 with or without a broad CLDN binder, i.e., C-terminal fragment of *Clostridium perfringens* enterotoxin (C-CPE), which can bind several CLDNs such as CLDN3, CLDN6, CLDN7, and CLDN9^17,18^, infection was completely blocked by C-CPE (Supplementary Fig. S7). These results strongly suggested that some CLDN family proteins other than CLDN1 are involved in its CLDN1-independent infection.

**Figure 4:**
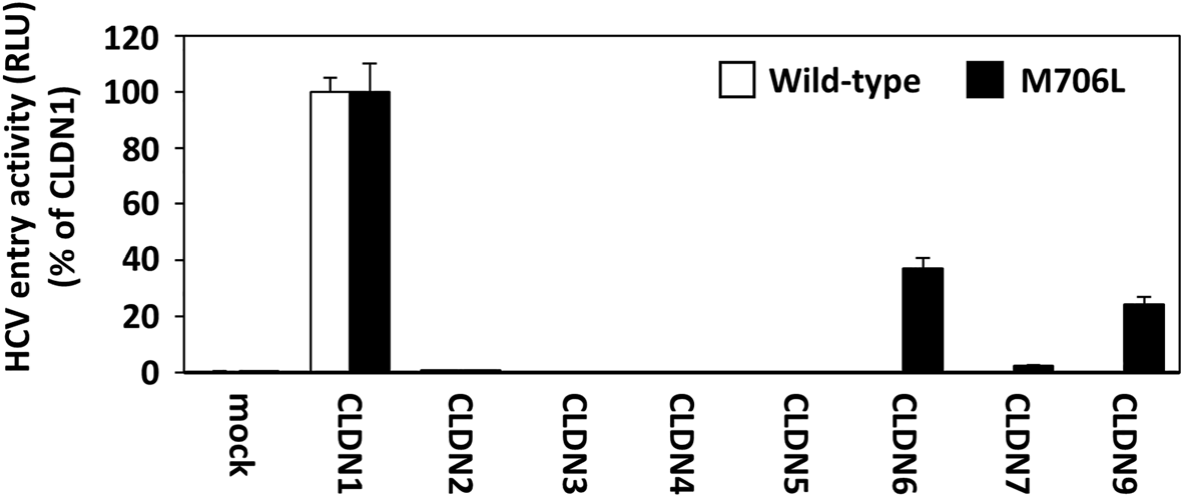
HCV-JFH1-based HCVpp carrying M706L mutation can infect HEK293T cells via CLDN1, CLDN6, and CLDN9-dependent pathways. HEK293T cells, transfected with mock or each CLDN, were infected with JFH1-based HCVpp with no (Wild-type; white) or M706L mutation (black) for 6 h. Two days p.i., luciferase activities of cell lysates were measured using a luminometer. Values were expressed as percentages of each value of CLDN1-expressing cells. Data are presented as the mean ± S.D. (n = 3).

We then explored the dependency of HCVpp with M706L mutation on various CLDN family proteins. Human embryonic kidney 293 T (HEK293T) cells stably expressing either CLDN1, CLDN2, CLDN3, CLDN4, CLDN5, CLDN6, CLDN7, or CLDN9 were infected with HCV-JFH1-based HCVpp without mutation (wild-type) or with M706L mutation. In Fig. 4, wild-type HCVpp can infect only CLDN1-expressing HEK293T cells, while HCVpp with M706L mutation can infect CLDN6- and CLDN9-expressing HEK293T cells as well as CLDN1-expressing cells. These results exhibited that HCVpp with M706L mutation can use CLDN6 and CLDN9 as an additional receptor.

**Figure 5:**
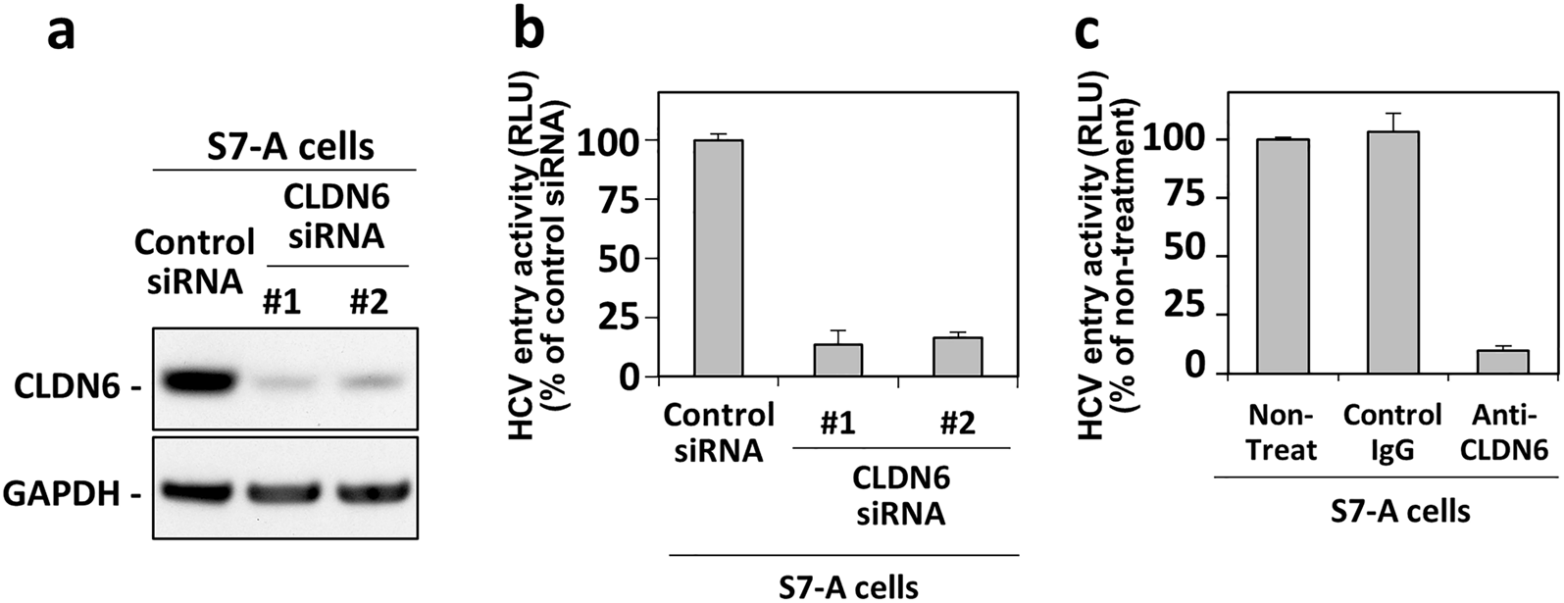
HCV-JFH1-based HCVpp carrying M706L mutation was capable of infecting CLDN1-defective S7-A cells in the CLDN6-dependent manner. (**a**, **b**) S7-A cells plated in 48-well plates were transfected with 25 nM of control siRNA or two types of siRNAs against CLDN6 and culture for 2 days. (**a**) Cells were lysed, and each cell lysate was subjected to immunoblotting for CLDN6 and GAPDH proteins. (**b**) Cells were infected with HCV-JFH1-based HCVpp having M706L mutation and culture for 2 days. (**c**) S7-A cells plated in 48-well plates were pretreated with 5 μg/ml of anti-CLDN6 mAb for 30 min, infected with HCV-JFH1-based HCVpp having M706L mutation, and cultured for 2 days. (**b**, **c**) Luciferase activities of cell lysates were then measured using a luminometer. Values were expressed as percentages of each control value (in (**b**), cells with control siRNA; and (**c**), cells without antibody treatment). Data are presented as the mean ± S.D. (n = 3).

In Huh7.5.1-8 cells, CLDN6 is expressed at the approximately 20% levels of CLDN1, but CLDN9 is expressed much less (< 1/10,000) than CLDN1 (Supplementary Table S2). CLDN1-defective S7-A cells have similar levels of CLDN6 and CLDN9 as Huh7.5.1-8 cells (Supplementary Table S2). Thus, CLDN6 is more likely than CLDN9 to contribute to M706L-carrying HCV-JFH1 infection in hepatic Huh7-derived cells. Indeed, infection with HCV-JFH1-based HCVpp having M706L mutation was prevented by more than 75% when CLDN6 was knocked down in S7-A cells by two types of CLDN6 siRNAs (Figs. 5a and 5b). Consistently, the treatment of S7-A cells with anti-CLDN6 antibodies resulted in more than 80% inhibition of infection with HCV-JFH1-based HCVpp with M706L mutation (Fig. 5c). In other hepatic cell line HUH-6 cells, CLDN6 is expressed more than tenfold higher than CLDN1, and the level of CLDN1 in HUH-6 cells is more than tenfold lower than that in Huh7.5.1-8 cells (Supplementary Table S2). Cell surface expression patterns of these CLDNs were also confirmed by flow cytometry analysis (Supplementary Fig. S8a). After infection with HCV-JFH1-tau or HCV-JFH1-tau Lot B1, viral production levels of both strains in Huh7.5.1-8 cells and the culture supernatants were similar, as already shown above in Supplementary Figs. S1a and S1b (white bars). Conversely, viral production levels of HCV-JFH1-tau Lot B1 in HUH-6 cells and the culture supernatants were significantly higher than those of HCV-JFH1-tau (Supplementary Figs. S8b and S8c). These results also agree with the finding that CLDN6 is involved in M706L-carrying HCV-JFH1 infection.

### M706L-carrying HCV-JFH1 can infect non-hepatic iPS cells

**Figure 6:**
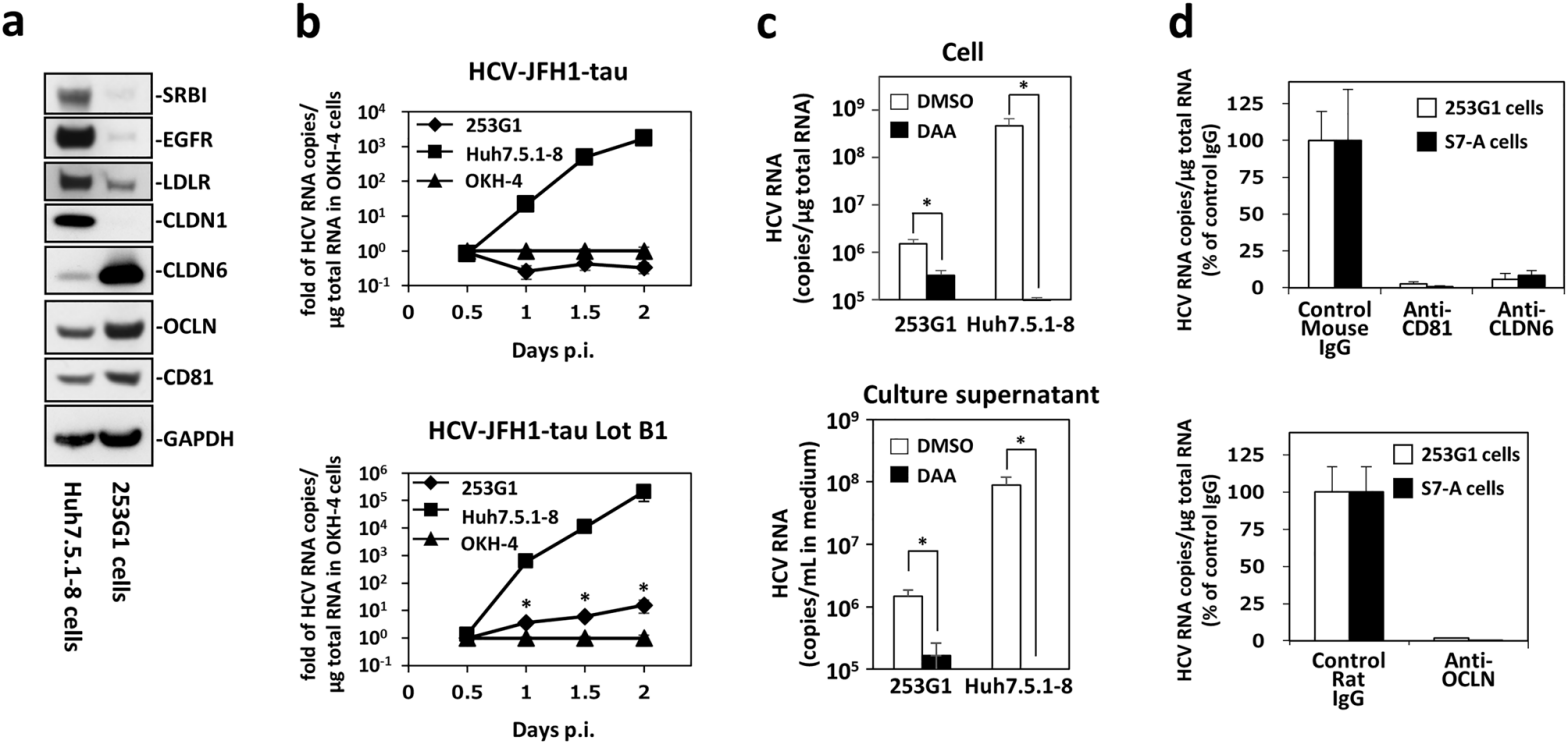
HCV-JFH1-tau Lot B1 having M706L mutation can infect non-hepatic iPS cells via the CLDN6-dependent pathway. (**a**) Cellular expression patterns of various HCV entry factors. Huh7.5.1-8 and 253G1 cells were lysed, and equal protein amounts of each cell lysate (10 μg) were subjected to immunoblotting for the SRBI, EGFR, LDLR, CLDN1, CLDN6, OCLN, CD81, and GAPDH proteins. (**b**) Huh7.5.1-8 (squares), OKH-4 (triangles), and 253G1 (diamonds) cells were infected with HCV-JFH1-tau or HCV-JFH1-tau Lot B1 at 1.0 × 10^5^ Geq/cell. At 0.5, 1, 1.5, and 2 days p.i., cellular HCV RNA contents were measured by qRT-PCR. Values are expressed as the ratio of OKH-4 at each day p.i. Data are presented as the mean ± S.D. (n = 6). *, *p *< 0.01 (vs. values of OKH-4 cells at each time point; Student’s *t* test). (**c**) 253G1 cells and Huh7.5.1-8 cells were infected with HCV-JFH1-tau Lot B1 at 1.0 × 10^5^ Geq/cell in the presence of DMSO (white) or 1 μM sofosbuvir, a direct-acting antiviral agent (DAA, black). At 2 days p.i., HCV RNA contents in cells and culture supernatants were measured by qRT-PCR. Data are presented as the mean ± S.D. (n = 10 in 253G1 and n = 6 in Huh7.5.1-8). *, *p *< 0.01 (vs. values of DMSO treated cells; Student’s t test). (**d**) 253G1 cells (white) and S7-A cells (black) were preincubated with 5.0 μg/ml of control mouse IgG, or 5.0 μg/ml of anti-CD81 mAb (clone JS-81), 5.0 μg/ml of anti-CLDN6 mAb (clone 342927), 3.0 μg/ml of control rat IgG, or 3.0 μg/ml of mAb against OCLN (clone 1–3) for 30 min at room temperature and then infected with HCV-JFH1-tau Lot B1 at 1.0 × 10^5^ Geq/cell. At 4 days p.i., cellular HCV RNA contents were measured by qRT-PCR. Values are expressed as the percentage of control mouse or rat IgG. Data are presented as the mean ± S.D. (n = 6).

CLDN6 is expressed mainly in stem cells, such as induced pluripotent stem (iPS) cells^19,20^. We also verified that 253G1 cell, a human iPS cell line established from adult dermal fibroblast, had a very high level of CLDN6 protein, compared with Huh7.5.1-8 cells (Fig. 6a). In contrast, 253G1 cells lacked the expression of CLDN1 protein, essential for general HCV infection. Other host proteins involved in HCV infection: SRBI, EGFR, low-density lipoprotein receptor (LDLR), OCLN, and CD81 were found in 253G1 cells, even though the expression levels of SRBI, EGRF, and LDLR were significantly lower than Huh7.5.1-8 cells (Fig. 6a).

We then addressed whether HCV-JFH1-tau Lot B1 with M706L mutation can infect iPS cells. HCV-JFH1-tau or HCV-JFH1-tau Lot B1 were inoculated into 253G1 cells, Huh7.5.1-8 cells, and OKH-4 cells, and cellular HCV RNA contents at 0.5–2 days p.i. were determined by qRT-PCR. Both HCV-JFH1-tau and HCV-JFH1-tau Lot B1 contents were time-dependently increased in the positive control Huh7.5.1-8 cells but not in the negative control OKH-4 cells (Fig. 6b). Interestingly, the HCV-JFH1-tau contents were background levels in 253G1 cells, but HCV-JFH1-tau Lot B1 contents in 253G1 cells were significantly high compared with those in the negative control OKH-4 cells, although the viral RNA levels in 253G1 cells were much lower than in Huh7.5.1-8 cells (Fig. 6b and supplementary Fig. S9). To further investigate whether HCV-JFH1-tau Lot B1 replicates in 253G1 cells, we examined the effect of HCV replication inhibitor, sofosbuvir, on HCV-tretaed 253G1 cells. As shown in Fig. 6c, HCV-JFH1-tau Lot B1 RNA contents in cells and culture supernatants were significantly reduced by sofosbuvir treatment in HCV-treated 253G1 cells as well as in HCV-tretated Huh7.5.1-8 cells. These results indicated that that HCV-JFH1-tau Lot B1 can infect and replicate in iPS cells. Consistent with these results, HCV-JFH1-based HCV pseudovesicles (HCVpv) with M706L mutation could infect iPS cells, but HCV-JFH1-based HCVpv with wild-type envelopes could not (Supplementary Fig. S10). Furthermore, infection of 253G1 cells with HCV-JFH1-tau Lot B1 having M706L mutation was blocked by anti-CLDN6 antibody as well as anti-CD81 and anti-OCLN antibodies (Fig. 6d). These results demonstrated that non-hepatic iPS cells could be infected with HCV-JFH1 substrain with M706L mutation via a CLDN6-dependent manner.

## Discussion

In this study, we repeatedly passaged HCV-JFH1-tau, which resultantly showed infectivity on CLDN1-defective S7-A cells (Fig. 1). And then, it was demonstrated that the M706L mutation in HCV E2 protein allows HCV-JFH1-tau to infect S7-A cells (Figs. 2 and 3). Whereas HCV-JFH1-tau infection depends only on CLDN1, HCV-JFH1-tau/M706L infection exhibited the expanded receptor usage options: CLDN1, CLDN6, and CLDN9 proteins (Figs. 4 and 5). Furthermore, the stem cells such as iPS cells are highly expressing CLDN6^20^, and iPS cells 253G1 were permissive to HCV-JFH1-tau Lot B1 having M706L mutation (Fig. 6).

What caused the emergence of HCV-JFH1-tau Lot B1 with broad CLDN selectivity? HCV infection of Huh-7 hepatoma cells results in the downregulation of CLDN1 and OCLN expression for preventing superinfection^21^. We now conceived that reducing cell surface CLDN1 after HCV infection has a selective advantage for the survival of HCV-JFH1-tau substrain with M706L mutation.

In Japan, we need a government (ministerial) permission to construct the whole genome of artificially modified viruses, that takes a very long time; and artificial modifications of viral genome to broaden the host ranges, which may enhance pathogenicity, are hard to be permitted ethically. That is why we did not perform the experiments using recombinant HCV-JFH1-tau M706L mutant in this study. Instead of these, we first tried to construct HCVpp infection system, but we could not produce infectious HCV-JFH1-tau Lot B1-type HCVpp, which has multiple mutations. Then, we developed the HCVee system (Supplementary Fig. S4A), which is an infection system using single-round infectious particles having various mutations in the envelope proteins. HCVee system worked very well, even in HCV construct with multiple mutations such as HCV-JFH1-tau Lot B1-type (Supplementary Fig. S4B). Although wild-type proteins as well as exogeneously expressed mutant proteins are contained in HCVee populations, the results using HCVee system were fully consistent with those using other HCVpp and HCVpv system, in which only one kind set of envelope proteins are contained. Thus, in this study we used the HCVee as a recombinant HCV infection system without modifications of viral genome, reflecting live HCV infection (Fig. 2 and Supplementary Fig. S5).

HCV-JFH1-tau Lot B1, with a reversed A392V mutation in the HCV E2 protein, lost the CLDN1-independent infection phenotype (Supplementary Fig. 5), and HCV-JFH1pp with only V392A point mutation did not show a CLDN1-independent infection (Fig. 3a). Therefore, although further investigations are needed, we suppose that the V392A mutation is involved in but not enough for the CLDN1-independent infection phenotype of HCV-JFH1-tau Lot B1, and additional mutations are necessary. Conversely, introducing a single M706L point mutation into HCV-JFH1 leads to acquiring a CLDN1-independent infection phenotype (Fig. 3 and Supplementary Fig. S5).

CLDN1, CLDN6, and CLDN9 usages on HCVpp infection were previously reported in other genotypes than genotype 2a, which HCV-JFH1 belongs to^22,23^. CLDN1 and CLDN6 usages on HCV-JFH1-based infectious chimeras with other genotypes (1a, 1b, 2b, 3a, 4a, and 6a) were also shown^24^. These results indicated the isolate-dependent use of CLDNs for cell entry by HCV. The introduction of the H316N mutation in the HCV E1 protein into HCV-J6 (genotype 2a) leads to the acquisition of CLDN1, 6 and 9-dependent infection^25^. Interestingly, HCV-JFH1-tau Lot B1 has the same H316N mutation (Table 1). However, the H316N mutation in HCV-JFH1-tau Lot B1 was unnecessary for CLDN1-independent infection (Supplementary Fig. 5 and Table 2). These results suggested that amino acid residues important for CLDN dependence in HCV infection are different between strains. We further tested the effects of M706L mutation on CLDN6 usage in various HCV strains (J6 (genotype 2a), H77 (genotype 1a), TH (genotype 1b)) as well as HCV-JFH1 (genotype 2a) using pseudoptype HCV infection system. First, we tried to introduce M706L mutation into another genotype 2a strain, HCV-J6. HCVpp-JFH1/M706L infected HEK293T/CLDN6, but HCVpp-J6/M706L did not (Supplementary Fig. S11b), suggesting that the effect of M706L mutation is strain-dependent. Next, we examined the effect in other genotype viruses: H77 (genotype 1a) and TH (genotype 1b). Both viruses have leucine at the position corresponding to aa706 of HCV-JFH1 (Supplementary Fig. S11a; aa702 of H77 and aa703 of TH), and these wild-type HCVpps infected HEK293T/CLDN6 (Supplementary Fig. S11c) as reported previously^12^. On the other hands, HCVpp-H77/L702M and HCVpp-TH/L703M infected HEK293T/CLDN1 and Huh7.5.1-8 cells, but did not infect HEK293T/CLDN6 (Supplementary Fig. S11c), indicating that this leucine residue is essential for acquitision of the expanded receptor usage of CLDN6 in H77 and TH srtrains of HCV.

HCV E2 glycoprotein interacts with CD81 and SRBI via its N-terminal domain^7,26,27^, but the region of HCV E2 that interacts with CLDN1 is unknown. M706 in HCV-JFH1 E2 is located close to the heptad repeat region (residue 675–699) that interacts with the HCV E1 protein^28^. In the previous report, the introduction of mutations into L675, S678, L689, and L692 residues in the heptad repeat of the E2 protein did not alter its CD81 binding significantly. However, it revealed the inability of E1E2-heterodimerization and a defect at a post-CD81 binding stage of entry^14,28^. Consistently, M706L-having HCV mutants still keep the dependency of SRBI and CD81 involved in the earlier stages of entry than CLDN1 (Supplementary Fig. S6). In the future, it is necessary to obtain the 3D structures of the E1-E2 complex and CLDNs for understanding the detailed receptor-dependent HCV entry mechanisms.

In large cohort studies, two-thirds of patients with HCV infection experienced extra-hepatic manifestations^29^. HCV-associated all-cause mortality is double compared to HCV-negative individuals, and extra-hepatic manifestations represent a major risk factor^30,31^. Various diseases are known as extra-hepatic lesions associated with HCV infection: lymphoproliferative disorders such as lymphadenopathy, splenomegaly, and membranoproliferative glomerulonephritis, and other diseases such as type 2 diabetes mellitus, sicca syndrome, and porphyria cutanea tarda^32–35^. Since hepatocyte-specific miR-122 is crucial for HCV replication, HCV genomic RNA can replicate efficiently in the liver. However, suppose cells from other organs have all of the host entry factors for HCV. In that case, HCV-derived polypeptides can be translated from the genomic RNA taken up via the endocytic pathway. At least four HCV proteins (core, NS3, NS5A, and NS5B) seem to play roles in several potentially oncogenic pathways^36–43^: for example, HCV core-transgenic mice develop symptoms of hepatocellular carcinoma. Among non-hepatic cells, stem cells in various tissues are known as cells strongly expressing CLDN6 but not expressing CLDN1^20^. We demonstrated that HCV-JFH1-tau Lot B1 having M706L mutation could infect iPS cells in the CLDN6-dependent manner (Fig. 6). Of note, only a single mutation can cause this interesting phenotypic change. Patients chronically infected with HCV have a large population of HCV variants termed quasispecies, likely including a single M706L mutation. Stem cells have well-proliferative and differentiable characteristics. Long-term attacks of M706L-having substrains to stem cells could last in those patients, and viral oncogenic and bioactive proteins such as core, NS3, NS5A, and NS5B, produced in the cells could be involved in HCV-related extra-hepatic lesions. There is very little possibility that the surface glycoproteins of viral particles and the packaged genomic RNA are the same genetic code (mutations), and HCV replication activity in stem cells would be much lower than that in hepatocytes (Fig. 6). Thus, proving the above-described hypothesis for the origins of extra-hepatic lesions would be very difficult. Early treatments against HCV infection might be needed to avoid the development of HCV-derived extra-hepatic lesions. The HCV-JFH1-tau substrain with broad CLDN selectivity would also be useful for pathological studies on cells other than hepatocytes.

## Methods

### Cells and cell culture

The Huh7.5.1-8 cell, derived from the Huh7.5.1 cell^44^, is a human hepatoma cell line highly permissive to HCV infection^45^. Huh7.5.1-derived CD81-defective 751r cells and CLDN1-defective S7-A cells, and Huh7.5.1-8-derived OCLN-knockout OKH-4 cells, all are non-permissive to HCV infection^9,11,45^. HEK 293 T cells were obtained from the American Type Culture Collection (Manassas, VA, USA). These cells were maintained at 37 °C and 5% CO_2_ in Dulbecco’s modified Eagle’s medium that contained 10% fetal bovine serum, 0.1 mM non-essential amino acids, 100 units/ml penicillin G, and 100 μg/ml streptomycin sulfate. In addition, human iPS cells (253G1-hiPSCs) were obtained from RIKEN BioResource Research Center. The methods used to culture were performed as described previously^46^. Stable transfectants of various CLDN families derived from HEK293T cells were established by retroviral-mediated gene transfer and drug selection, as described previously^11^.

### Reagents and antibodies

MTT was obtained from Sigma (St. Louis, MO). DAPI was purchased from Life Technologies (California, USA). Validated stealth siRNAs of CLDN6 (HSS144989, HSS189836) and negative control siRNA (Stealth RNAi Negative Control Duplex Low GC Duplex #2) were purchased from Life Technologies (California, USA). Mouse mAb against the HCV core protein (clone 2H9) was described previously^47^. Mouse control IgG was purchased from Southern Biotech (Alabama, USA). Mouse mAb against CLDN1 (clones 2C1) was described previously^9^. Rabbit polyclonal antibodies against CLDN6 protein for immunoblot analysis were purchased from Immuno-Biological Laboratories (Gunma, Japan). Mouse mAb against glyceraldehyde 3-phosphate dehydrogenase (GAPDH; clone 6C5) was purchased from Wako Pure Chemical Industries (Tokyo, Japan). Mouse mAb against CLDN6 (clone 342927), used in HCV infection assays, was purchased from R&D Systems (Minneapolis, MN, USA). Rabbit mAb against SRBI (clone EP1556Y) was purchased from Abcam (Cambridge, UK). Rabbit polyclonal antibodies against EGFR were purchased from Cell signaling (Massachusetts, USA). Mouse mAb against the LDLR (clone 15C8) was purchased from EMD Millipore (Billerica, MA, USA). Rabbit polyclonal antibodies against CLDN1 and mouse mAb against OCLN (clone OC-3F10) were purchased from Life Technologies (California, USA). Mouse mAb against CD81 (clone JS-81) was purchased from BD Bioscience Pharmingen (San Jose, CA, USA). Rat control IgG was purchased from Jackson ImmunoResearch Laboratories, Inc. (West Grove, PA, USA). Rat mAb against OCLN (clone 1–3) was described previously^12,13,48^. Sofosbuvir was purchased from Cayman CHEMICAL (Ann Arbor, MI, USA).

### Pseudoparticles derived from mouse leukemia virus

HCV and vesicular stomatitis virus (VSV) pseudo particles derived from mouse leukemia virus (HCVpp and VSVpp) were generated as described previously^49^. Briefly, a Gag–Pol packaging construct (Gag–Pol 5349), a transfer vector construct (Luc 126), and an envelope glycoprotein (E1 and E2)-expressing vector pcDNA3.1(+)-HCV-JFH1 ΔC-E2 [JFH1, genotype 2a (GenBank accession number AB047639.1); or VSV-G (GenBank accession number M27165)] were transfected into HEK 293 T cells. The medium from transfected cells was collected and used as the HCVpp and VSVpp sources. Pseudoparticles infection and luciferase reporter assays were performed as described previously^45^. The pcDNA3.1(+)-HCV-JFH1-ΔC-E2 plasmids with mutations were prepared by the inverse PCR methods described previously^11^.

### Production of HCV encapsidated with exogeneous envelope proteins (HCVee)

Huh7.5.1-8 cells were seeded at a density of 5 × 10^4^ cells/well in 24-well plates and incubated at 37 °C overnight. Cells were then infected with HCV-JFH1-tau at a MOI of 1 and cultured for 2 days. Infected cells were transfected with 0.5 μg of each plasmid (pcDNA3.1(+)-HCV-JFH1-ΔC-E2 wild-type or mutants) using 2 μl of X-tremeGENE HP DNA Transfection Reagent (Roche Diagnostics, Basel, Switzerland), following the manufacturer’s instruction. After 3 days the culture supernatant that contained HCVee egressed from these infected cells, were collected and centrifuged to remove cell debris. Naive cultured cells were seeded one day before infection and infected with these HCVee. Three days p.i. HCVee infectivity was determined by immunohistochemistry using anti-HCV core protein mAb clone 2H9. The pcDNA3.1(+)-HCV-JFH1-ΔC-E2 plasmids with mutations were prepared by the inverse PCR methods described previously^11^.

### Other reagents and methods

MTT assays were performed as described previously to determine cell viability^50^. The HCV strain used in this study is of HCV-JFH1 origin. The HCV-JFH1 strain (genotype 2a), originally cloned from an individual with fulminant hepatitis, can infect cultured hepatic cells^47^. HCV-JFH1-tau, adapted to Huh7.5.1 cells, is a highly infectious substrain of HCV-JFH1^16^. Collection and purification of total cellular RNA, quantification of HCV RNA copies, luciferase assay, and immunohistochemistry were performed as described previously^16^. The method of immunoblot analysis was described previously^45^. Nucleotide sequences of the HCV structural protein region were determined as described previously^16^. Briefly, overlapping cDNA fragments spanning the entire open reading frame of HCV-JFH1 structural protein region were amplified from the cellular total RNA using HCV-specific primer sets by one-step RT-PCR (QIAGEN). The resultant PCR products (several hundred base pairs) were subjected to direct sequencing. The numbering system for amino acids is based on polyprotein of HCV-JFH1.

## Supplementary Information

Supplementary Information.
