# Supplementary material for: A single mutation in the E2 glycoprotein of hepatitis C virus broadens the claudin specificity for its infection
Source: Sci Rep. 2022 Nov 24;12:20243. doi: 10.1038/s41598-022-23824-3 (PMC9691748; doi:10.1038/s41598-022-23824-3)
Supplement: Supplementary file 1 — Supplementary Information. [file 41598_2022_23824_MOESM1_ESM.pdf]

SUPPLEMENTARY INFORMATION

Cells and cell culture

HUH-6 cells clone 5 (JCRB0401, lot No. 03032014) were purchased from Japanese Collection of Research Bioresources (Osaka, Japan) and maintained at 37°C and 5% CO_2 in Dulbecco's modified Eagle's medium (DMEM) that contained 10% fetal bovine serum, 0.1 mM non-essential amino acids, 100 units/ml penicillin G, and 100 μg/ml streptomycin sulfate. Other cells were described in the Methods of the main text.

Reagents and Antibodies

Mouse mAb against HCV NS3 protein (clone 8G2) was purchased from Abcam (Cambridge, UK). Mouse mAbs against CLDN1 (clones 2C1 and 3A2) were described previously [1]. A broad CLDN binder, C-terminal fragment of the *Clostridium perfringens* enterotoxin (C-CPE) and its derivative C-CPE mutant (negative claudin binder, with Y306A/L315A substitutions), were described previously [2-4]. C-CPE binds to various CLDNs, such as CLDN3, CLDN6, CLDN7, and CLDN9. Other antibodies and reagents used were described in the Methods of the main text.

Negative control siRNA (Stealth RNAi Negative Control Duplex Low GC Duplex #2) was used. In addition, validated stealth siRNAs of scavenger receptor class B type I (SRBI) (HSS101570, HSS101571) were purchased from Life Technologies (California, USA).

Quantification of mRNA

SRBI, CLDN1, CLDN6, CLDN9, and GAPDH mRNA contents were determined using qRT-PCR with RNAdirect™ SYBR Green Realtime PCR Master Mix (Toyobo Co. Ltd.,

Osaka, Japan), and the specific primers for SRBI (5′-ATGAAATCTGTCGCAGGCATTG-3′ and 5′-TGCATCACCTTGGGCATCA-3′), CLDN1 (5′- CGGCGACAACATCGTGACC-3′ and 5′-AACCACCATCAAGGCACGG-3′), CLDN6 (5′-AATGCAGATCCTGGGAGTCGTC-3′ and 5′-ACGATGCTGTTGCCGATGAA-3′), CLDN9 (5′-TTCGACCTTGGCCTGATGAC-3′ and 5′-CTGCAGCCAGGTGTAGCTTG-3′), GAPDH (5′-GCACCGTCAAGGCTGAGAAC-3′ and 5′-TGGTGAAGACGCCAGTGGA-3′).

Pseudoparticles derived from recombinant vesicular stomatitis virus

Recombinant VSV (VSVΔG*) encodes the green fluorescent protein (GFP) gene instead of the glycoprotein (G) gene. HCV glycoprotein-coated or VSV-G-coated VSVΔG* was named as HCVpv or VSVpv, respectively. The methods of HCVpv and VSVpv production were as described [5]. Briefly, HEK293T cells were seeded at a density of 2×10^6 cells in 10 cm dish and incubated overnight. Cells were transfected with 10 μg of each plasmid (pcDNA3.1(+)-HCV-JFH1-ΔC-E2 wild-type or mutants) using 40 μg of the PEI Max (Cosmo Bio, Tokyo, Japan). Transfected cells were infected with VSVΔG* at a multiplicity of infection (MOI) of 1. The virus was adsorbed for 2 h at 37°C and washed extensively three times with DMEM without FBS. After 16 h of incubation at 37°C, culture supernatants were collected and centrifuged to remove cell debris. HCVpv and VSVpv infectivity were determined by observing GFP expressed by virus infection with a confocal microscope LSM800 (ZEISS, Oberkochen, Germany).

Other methods

The methods of flow cytometry and determination of HCV infectivity titers were

described previously [6]. Nucleotide fractions of culture supernatants were purified, and their HCV RNA contents were quantified by qRT-PCR as described previously [7]. Other HCV infection experiments and detection of HCV RNAs and proteins using qRT-PCR, immunoblot analysis, and fluorescent microscopy were described in the Methods of the main text. HCVee and HCVpp preparations and their infections were described in the Methods of the main text. Expression plasmids of each HCV strain (JFH1, J6, H77, and TH) using pseudoparticles infection experiments were described previously [8]. These plasmids with a point mutation were prepared by the inverse PCR methods described previously [7].

	FFU/10^8 copies of HCV RNA	
	HCV-JFH1-tau	
Cells	parent (original)	Lot B1
Huh7.5.1-8	$(1.5 \pm 0.6) \times 10^3$	$(1.0 \pm 0.5) \times 10^3$
S7-A (ΔCLDN1)	ND	$(1.3 \pm 0.7) \times 10^3$

ND, not detected. n = 3

Supplementary Table S1 Infectivity titers of HCV-JFH1-tau and HCV-JFH1-tau Lot B1 in Huh7.5.1-8 and CLDN1-defective S7-A cells

Cell line	copies/μg total RNA				
	SRBI mRNA	CLDN1 mRNA	CLDN6 mRNA	CLDN9 mRNA	GAPDH mRNA
Huh7.5.1-8	$(4.8 \pm 0.2) \times 10^4$	$(5.8 \pm 1.3) \times 10^5$	$(1.1 \pm 0.0) \times 10^5$	$(4.6 \pm 1.0) \times 10^1$	$(1.2 \pm 0.1) \times 10^8$
S7-A (ΔCLDN1)	$(7.0 \pm 0.3) \times 10^4$	ND ($<10^2$)	$(1.1 \pm 0.3) \times 10^5$	$(4.3 \pm 0.7) \times 10^1$	$(1.3 \pm 0.2) \times 10^8$
HUH-6	$(1.1 \pm 0.1) \times 10^4$	$(2.3 \pm 0.5) \times 10^4$	$(3.9 \pm 0.7) \times 10^5$	$(1.1 \pm 0.2) \times 10^2$	$(1.5 \pm 0.1) \times 10^8$

ND, not detected. n = 3

Supplementary Table S2 Expression levels of SRBI, CLDN1, CLDN6, CLDN9, and GAPDH in Huh7.5.1-8 cells, S7-A cells, and HUH-6 cells

Supplementary Figure Legends

Supplementary Fig. S1 HCV-JFH1-tau Lot B1 can infect CLDN1-defective S7-A cells.

Huh7.5.1-8, S7-A, or 751r cells were infected with HCV-JFH1-tau (**a**, **c**, **e**) or HCV-JFH1-tau Lot B1 (**b**, **d**, **f**) at 1.0×10^4 Geq/cell. One, two, and three days postinfection (p.i.), HCV RNA contents in supernatants (**a**, **b**) were measured by qRT-PCR (n = 4). Four days p.i., cell lysates were subjected to immunoblotting to detect HCV NS3, core, and a host house-keeping gene GAPDH (**c**, **d**), and cells were stained with anti-HCV core mAb (green) and DAPI (blue) and observed using fluorescence microscopy (**e**, **f**). Dashed lines in **a** and **b** are the limit of detection, values of which were $(5.94 \pm 1.43) \times 10^6$ copies/ml, calculated from the values of no infection. Bars in **e** and **f,** 50 μm.

Supplementary Fig. S2. Anti-CLDN1 antibodies did not inhibit HCV-JFH1-tau Lot B1 infection.

Huh7.5.1-8 cells were preincubated with 20 μg/ml of each control mouse IgG or anti-CLDN1 mAbs (clone 2C1 or 3A2) for 30 min at room temperature and then infected with HCV-JFH1-tau or HCV-JFH1-tau Lot B1 at 1.0×10^4 Geq/cell for 2 hours at 37°C. Cells were further cultured in the presence of each antibody. Three days p.i., cellular HCV RNA contents were measured by qRT-PCR (**a**), and cell lysates were subjected to immunoblotting to detect HCV core, NS3 and GAPDH (**b**). Values in **a** are expressed as percentages of control values (treatment with control IgG). Data are presented as means ± S.D. (n = 3).

Supplementary Fig. S3 HCV-JFH1-tau Lot B1 infection depends on occludin.

Huh7.5.1-8, S7-A, and Huh7.5.1-8-derived occludin (OCLN)-knockout OKH-4 cells were infected with HCV-JFH1-tau or HCV-JFH1-tau Lot B1 at 1.0×10^4 Geq/cell. Three days p.i., cellular HCV RNA contents were measured by qRT-PCR (**a**), and cell lysates were subjected to immunoblotting to detect HCV core, NS3 and GAPDH (**b**). Data are presented as means ± S.D. (n = 4). Dashed line in **a** is the detection limit, the value of which was $(5.75 \pm 1.11) \times 10^5$ copies/μg total RNA, calculated from the values of no infection.

Supplementary Fig. S4 HCVee infection system

(**a**) Preparation of HCV encapsidated with exogenously-supplemented HCV envelope proteins (HCVee) and its infection. Huh7.5.1-8 cells in a 24-well plate were seeded at 5×10^4 cells/well, cultured overnight, and infected at a multiplicity of 1 with HCV-JFH1-tau at 37°C. Two days p.i., infected cells were transfected with pcDNA3.1(+)-HCV-JFH1-ΔC-E2 wild-type or Lot B1-type plasmids at a weight of 0.5 μg by X-treme GENE HP DNA transfection reagent (Roche). The viral supernatants were collected 3 days after transfection, and one-half of the supernatant fluid was used to infect the second plate of naïve cells: Huh7.5.1-8, S7-A, and 751r cells. Three days p.i., cells were stained with anti-HCV core protein mAb (green) and DAPI (blue) and observed using fluorescence microscopy (**b**). Bars in **b**, 50 μm.

Supplementary Fig. S5 Mutation analysis using HCVee system

HCVee preparation and infection were performed as described in the Methods of the main text, in which pcDNA3.1(+)-HCV-ΔC-E2 Lot B1-type plasmids with each wild-type point mutation were used. HCVee-infected Huh7.5.1-8, S7-A, and 751r cells were

stained with anti-HCV core protein mAb (green) and DAPI (blue) and observed using fluorescence microscopy. Bars, 50 μm.

Supplementary Fig. S6 Infection of Huh7.5.1-8 cells with HCVpp carrying M706L mutation depends on CD81, SRBI, and OCLN.

(**a**, **b**) Huh7.5.1-8 cells plated in 48-well plates were transfected with control siRNA or two types of siRNAs against SRBI. (**a**) Two days after transfection, cells were lysed, and each cell lysate was subjected to immunoblotting for SRBI and GAPDH proteins. (**b**) Two days after transfection, cells were infected with HCV-JFH1-based HCVpp having no mutation (white) or M706L mutation (black). Two days p.i., luciferase activities of cell lysates were measured using a luminometer. Values were expressed as percentages of control values (treatment with control siRNA). Data are presented as the mean ± S.D. (n = 3). (**c**) Huh7.5.1-8, OKH-4, S7-A, and 751r cells were infected with HCV-JFH1-based HCVpp having no mutation (white) or M706L mutation (black), or VSVpp (dot) for 6 h. Two days p.i., luciferase activities of cell lysates were measured using a luminometer. Values were expressed as percentages of each value of Huh7.5.1-8 cells. Data are presented as the mean ± S.D. (n = 6).

Supplementary Fig. S7 Infection of S7-A cells with HCV-JFH1-tau Lot B1 was inhibited by broad CLDN binder C-CPE.

S7-A cells were preincubated with 20 μg/ml of each control mouse IgG or anti-CLDN1 mAb (2C1), and 5 μg/ml of C-CPE wild-type (WT) or mutant (M) for 30 min at room temperature and then infected with HCV-JFH1-tau Lot B1. Four days p.i., infected cells were lysed, and each cell lysate was subjected to immunoblotting for HCV NS3, HCV

core and GAPDH proteins, using TrueBlot anti-mouse IgG HRP as a secondary antibody (1:1,000, Rockland Immunochemicals, Inc., Gilbertsville, PA).

Supplementary Fig. S8 HCV-JFH1-tau Lot B1 can efficiently infect other hepatic HUH-6 cells, endogenously having higher CLDN6 but lower CLDN1 expression, compared with Huh7.5.1-8 cells

(**a**) Huh7.5.1-8 and HUH-6 cells were stained with no treatment (gray) and mAbs against CD81, CLDN1 and CLDN6 (white) and analyzed using flow cytometry. In bottom Table, +, 10<MFI (mean fluorescence intensity) <100; ++, 100<MFI<1000. (**b**, **c**) Huh7.5.1-8 and HUH-6 cells were infected with HCV-JFH1-tau or HCV-JFH1-tau Lot B1 at 1.0×10^4 Geq/cell. One, two, and three days p.i., cellular HCV RNA contents (**b**) and HCV RNA contents in supernatants (**c**) were measured by qRT-PCR. Data are presented as means ± S.D. (n = 4).

Supplementary Fig. S9 HCV-JFH1-tau Lot B1 having M706L mutation can infect non-hepatic iPS cells.

This figure is the raw data of Fig. 6**b** in the main text. Huh7.5.1-8 (squares), OKH-4 (triangles), and 253G1 (diamonds) cells were infected with HCV-JFH1-tau (**a**) or HCV-JFH1-tau Lot B1 (**b**) at 1.0×10^5 Geq/cell. At 0.5, 1, 1.5, and 2 days p.i., cellular HCV RNA contents were measured by qRT-PCR. Data are presented as the mean ± S.D. (n = 6). *, $p < 0.01$ (vs. values of OKH-4 cells at each time point; Student's *t* test).

Supplementary Fig. S10 M706L mutation in HCV-JFH1-based HCVpv is essential for its infection of non-hepatic iPS cells

253G1, Huh7.5.1-8, or OKH-4 cells were infected with GFP reporter-carrying VSVpv or HCV-JFH1-based HCVpv having no envelopes (-), no mutations (wild-type), or M706L mutation. One day p.i., cells were stained with DAPI and observed GFP (green) and DAPI (blue) using fluorescence microscopy. Bars, 50 μm.

Supplementary Fig. S11 Involvement of leusine residue in the acquisition of the expanded receptor usage of CLDN6 in various HCV strains.

(a) Comparison of amino acid sequences of E2 (aa661-720 in JFH1) among four HCV strains: JFH1, J6, H77, and TH. These sequence data are available from the NCBI database. Accession numbers: JFH1 (genotype 2a), AB047639.1; J6 (genotype 2a), AEJ90142.1, H77 (genotype 1a), JX472009.1; TH (genotype 1b), BAQ54173.1. Arrow, mutation site in JFH1/M706L. (b) M706L mutation is important for acquisition of the expanded receptor usage of CLDN6 in HCV-JFH1 strain, but not in HCV-J6 strain. HEK293T cells (mock) and HEK293T cells stably expressed CLDN1 or CLDN6 were infected with JFH1-based (black and gray) or J6-based (white and hatched) HCVpp with no (wild-type; black and white) or M706L mutation (gray and hatched) for 6 h. Two days p.i., luciferase activities (relative luminescence units: RLU) of cell lysates were measured using a luminometer. Data are presented as the mean ± S.D. (n = 3). (c) Leucines at aa702 of H77 strain and at aa703 of TH strain are important for acquisition of the expanded receptor usage of CLDN6. HEK293T (mock), HEK293T cells stably expressed CLDN1 or CLDN6, and Huh7.5.1-8 cells were infected with JFH1-based (black and gray), H77-based (white and hatched), or TH-based (dot and checkered) HCVpp with no (wild-type; black, white, and dot) or M706L (gray), L702M (hatched), or L703M (checkered)

mutation for 6 h. Two days p.i., luciferase activities (relative luminescence units: RLU) of cell lysates were measured using a luminometer. Data are presented as the mean ± S.D. (n = 3).

Supplementary References

1. Fukasawa, M. *et al.* Monoclonal antibodies against extracellular domains of claudin-1 block hepatitis C virus infection in a mouse model. *J Virol.* **89**, 4866-4879 (2015).
2. Kakutani, H. *et al.* A novel screening system for claudin binder using baculoviral display. *PLoS One.* **6**, e16611 (2011).
3. Takahashi, A. *et al.* Creation and biochemical analysis of a broad-specific claudin binder. *Biomaterials.* **33**, 3464-3474 (2012).
4. Takahashi, A. *et al.* Domain mapping of a claudin-4 modulator, the C-terminal region of C-terminal fragment of Clostridium perfringens enterotoxin, by site-directed mutagenesis. *Biochem Pharmacol.* **75**, 1639-1648 (2008).
5. Matsuura, Y. *et al.* Characterization of pseudotype VSV possessing HCV envelope proteins. *Virology.* **286**, 263-275 (2001).
6. Shirasago, Y. *et al.* Isolation and characterization of an Huh.7.5.1-derived cell clone highly permissive to hepatitis C virus. *Jpn J Infect Dis.* **68**, 81-88 (2015).
7. Shirasago, Y. *et al.* Occludin-Knockout Human Hepatic Huh7.5.1-8-Derived Cells Are Completely Resistant to Hepatitis C Virus Infection. *Biol Pharm Bull.* **39**, 839-848 (2016).
8. Shimizu, Y. *et al.* Monoclonal Antibodies against Occludin Completely Prevented Hepatitis C Virus Infection in a Mouse Model. *J Virol.* **92** (2018).

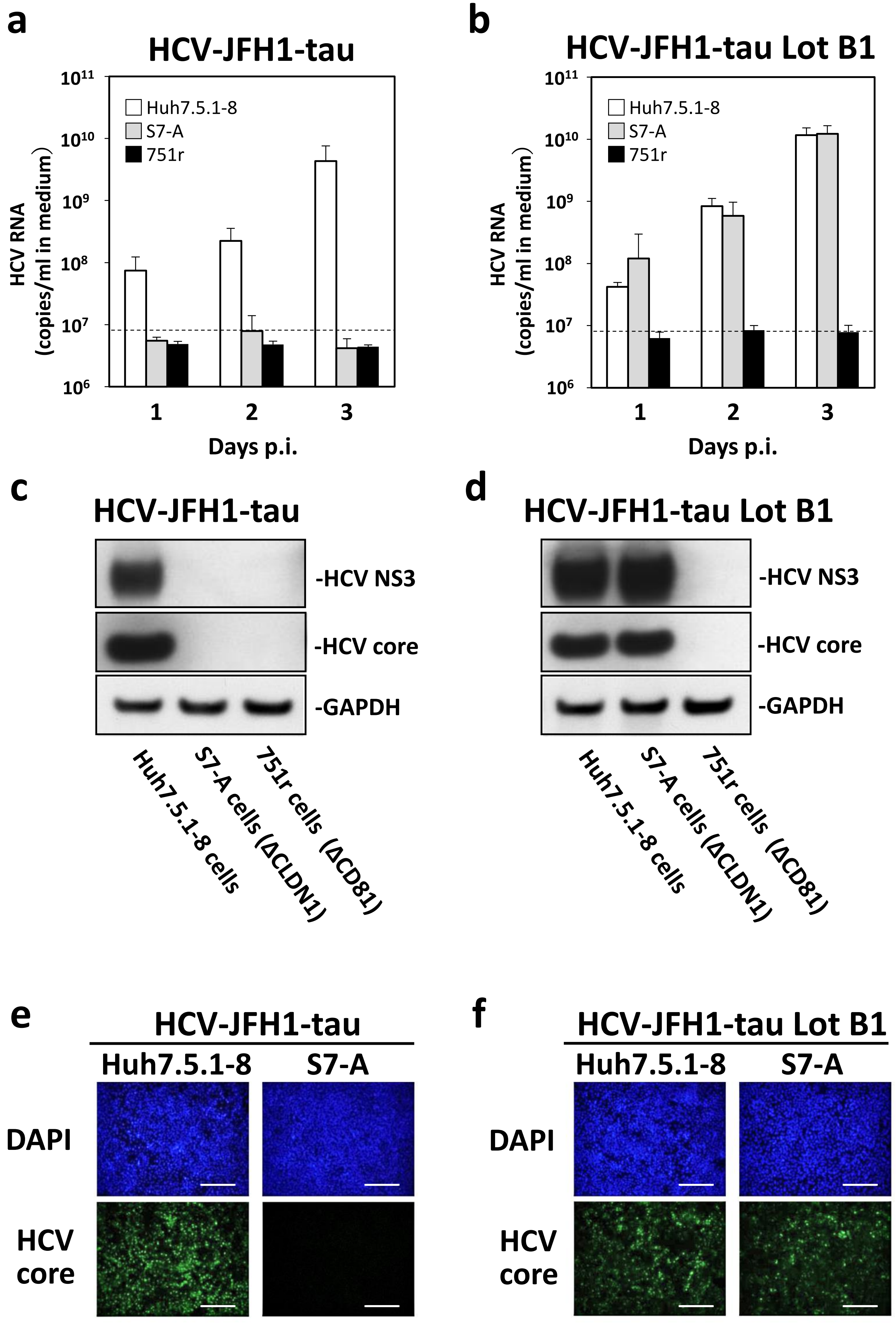

Supplementary Fig. S1

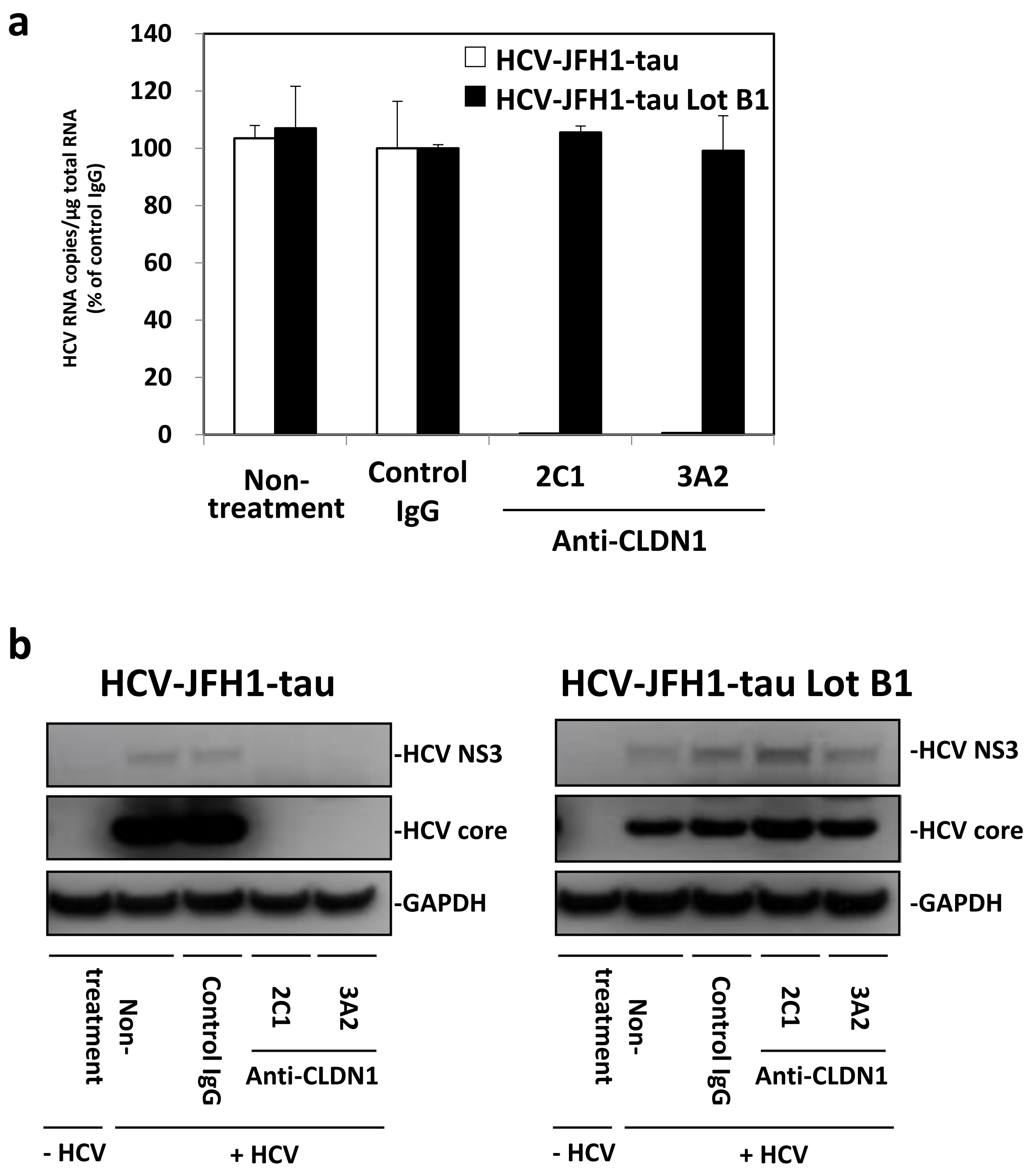

Supplementary Fig. S2

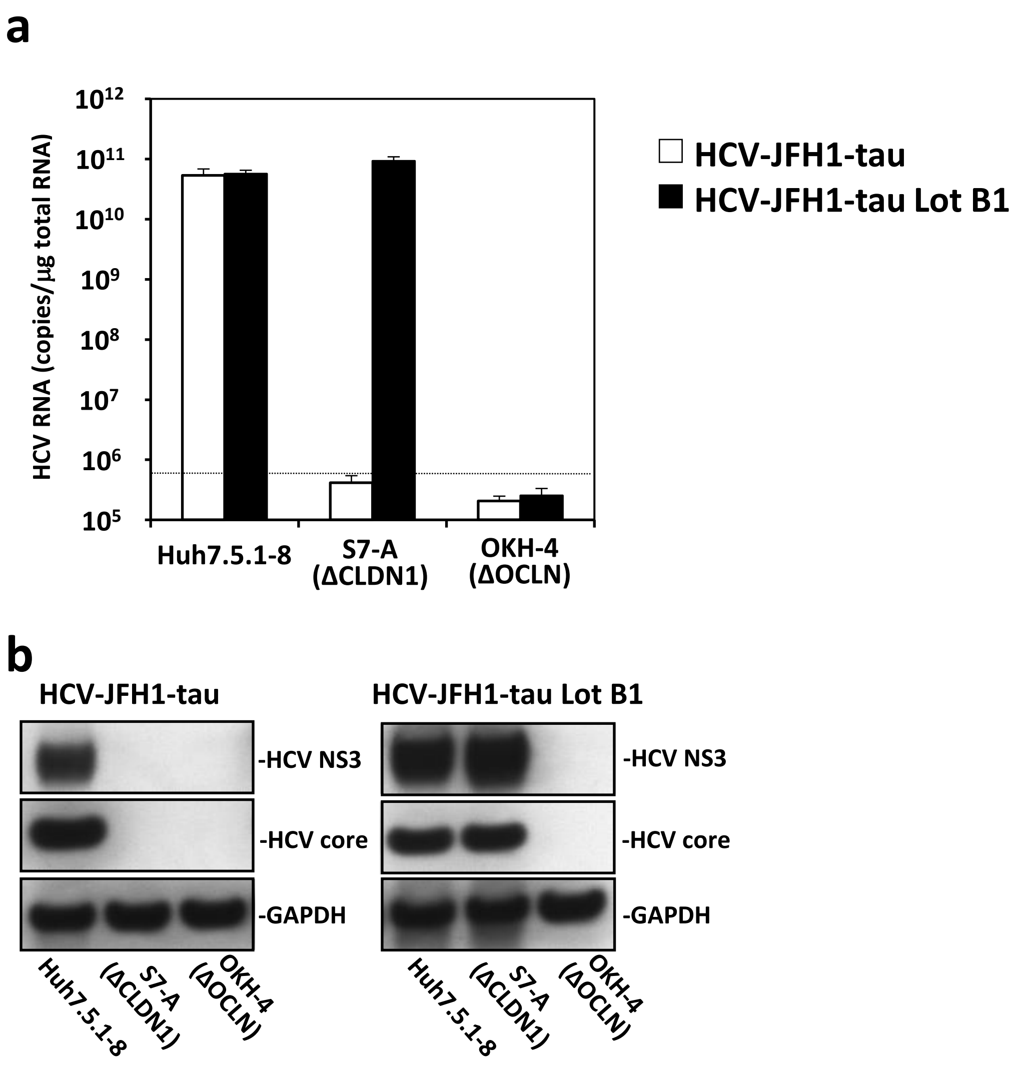

Supplementary Fig. S3

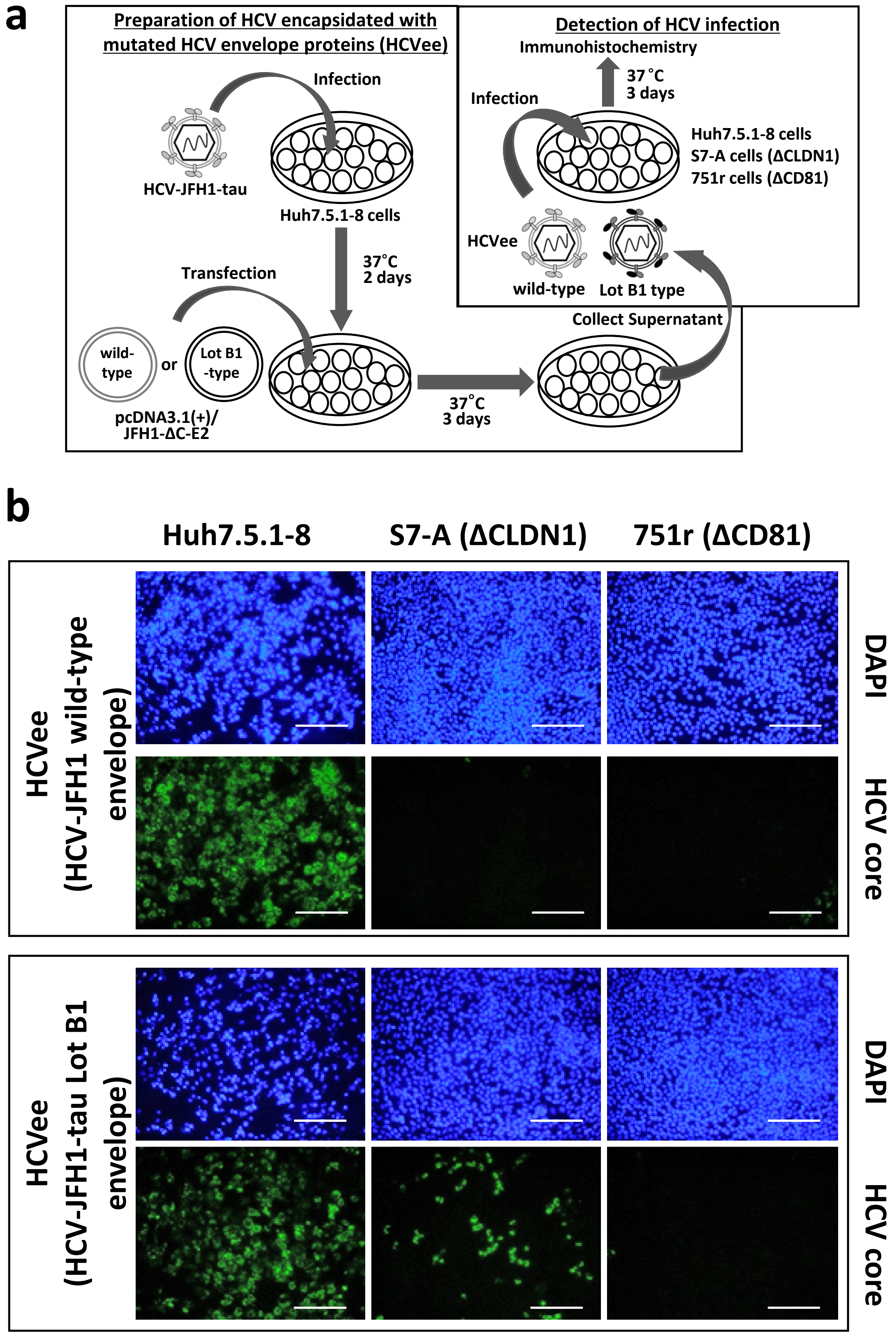

Supplementary Fig. S4

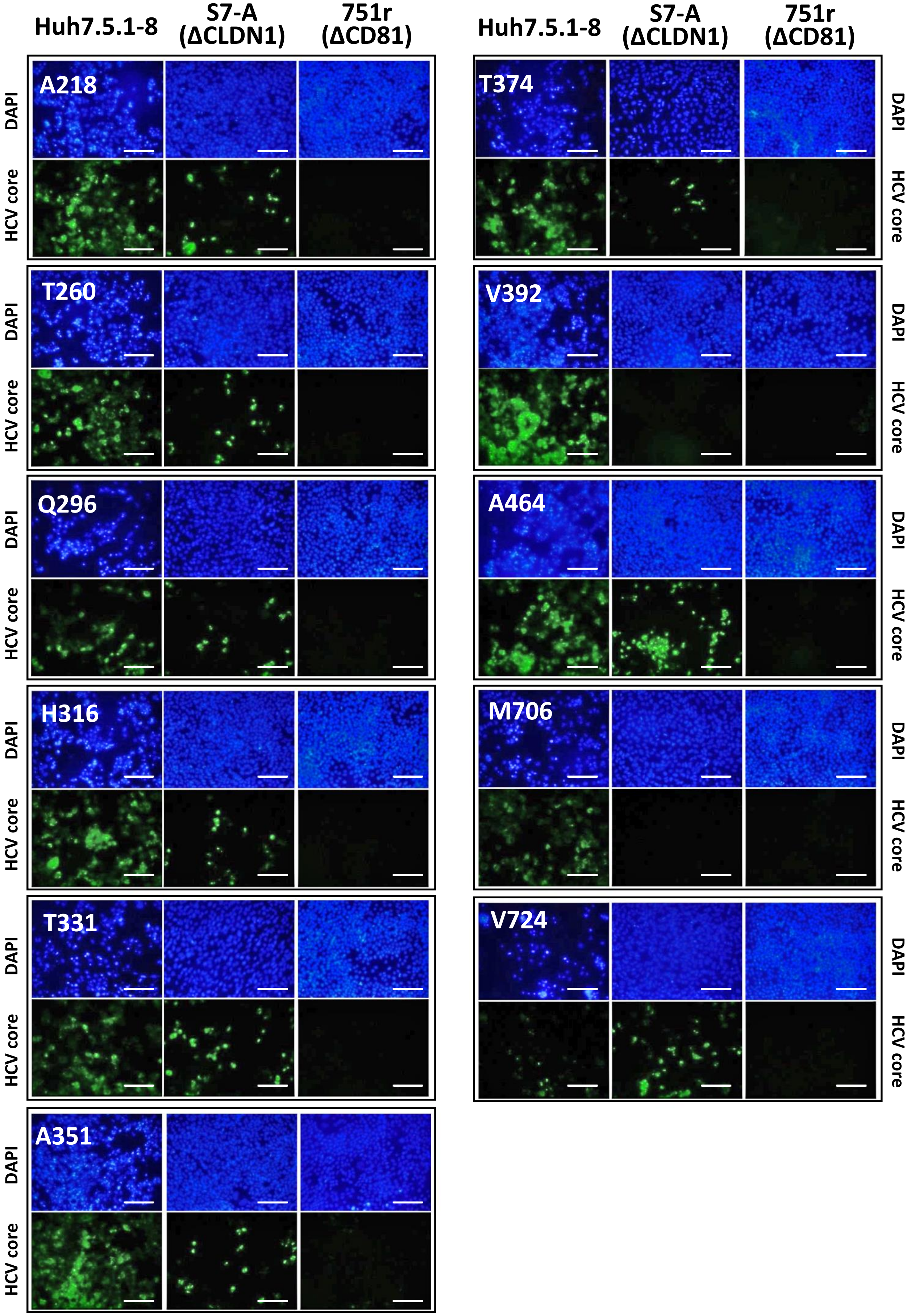

Supplementary Fig. S5

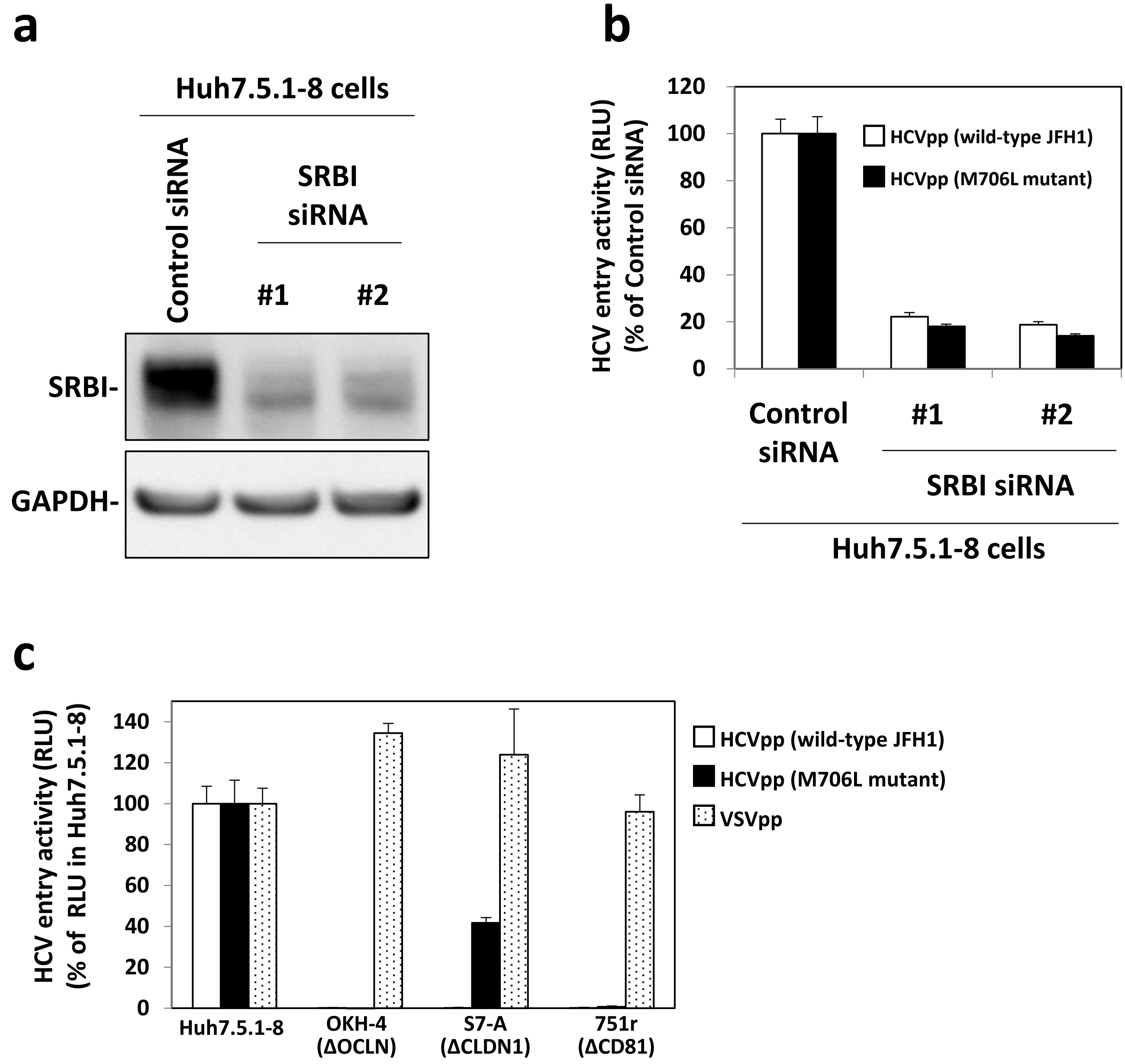

Supplementary Fig. S6

S7-A (ΔCLDN1) cells

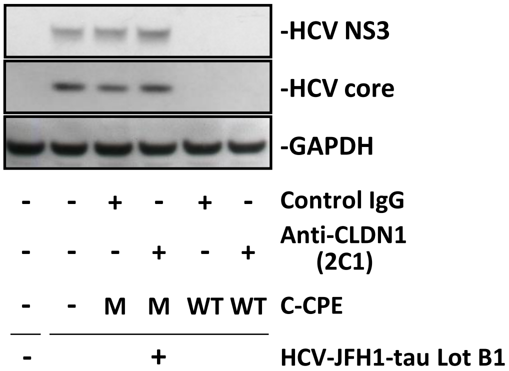

Supplementary Fig. S7

a

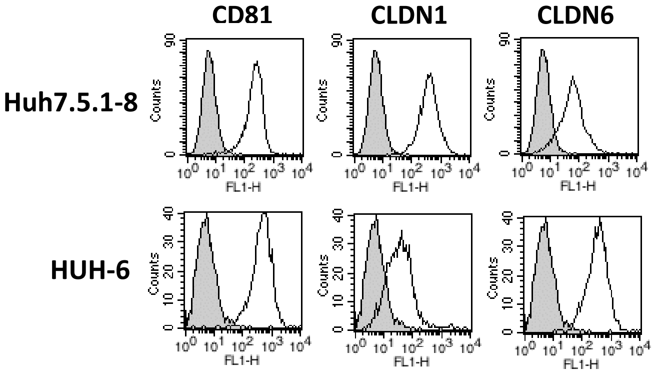

	CD81	CLDN1	CLDN6
Huh7.5.1-8	+	++	+
HUH-6	+	+	++

b

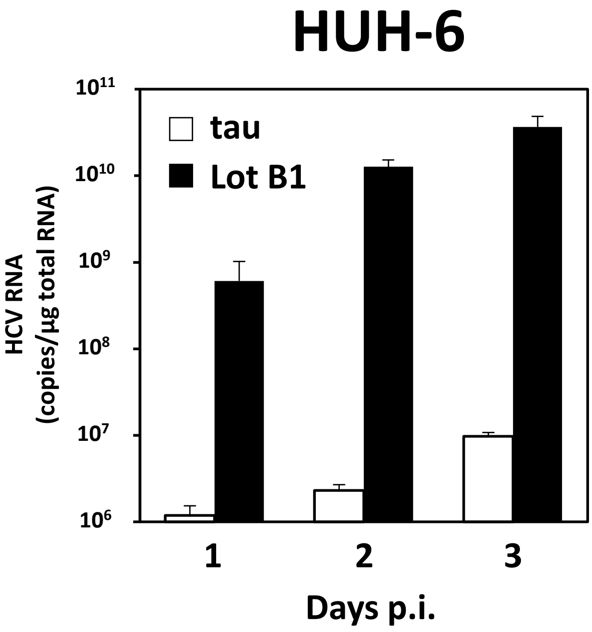

c

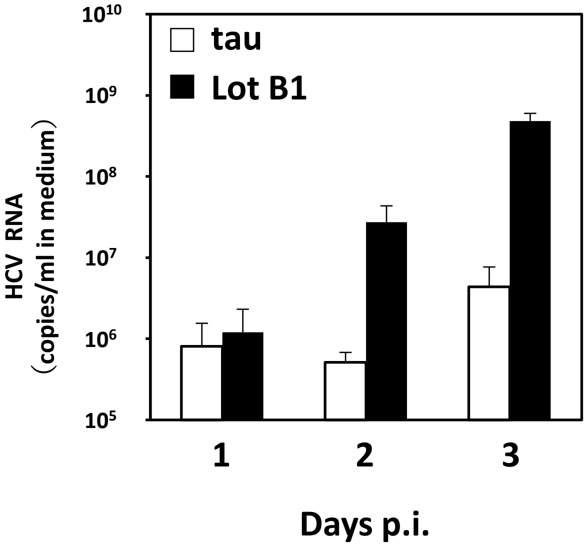

Supplementary Fig. S8

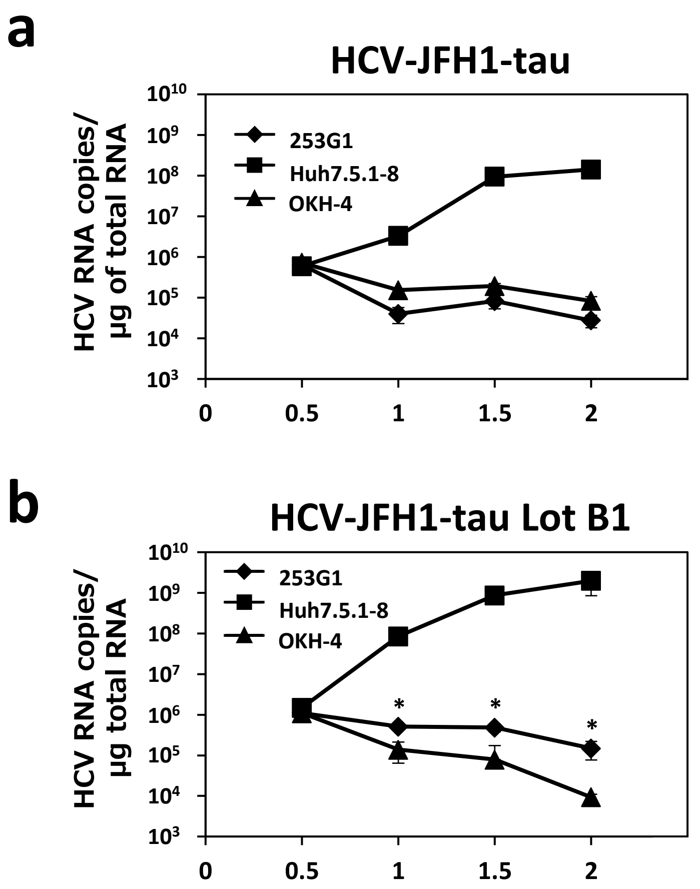

Supplementary Fig. S9

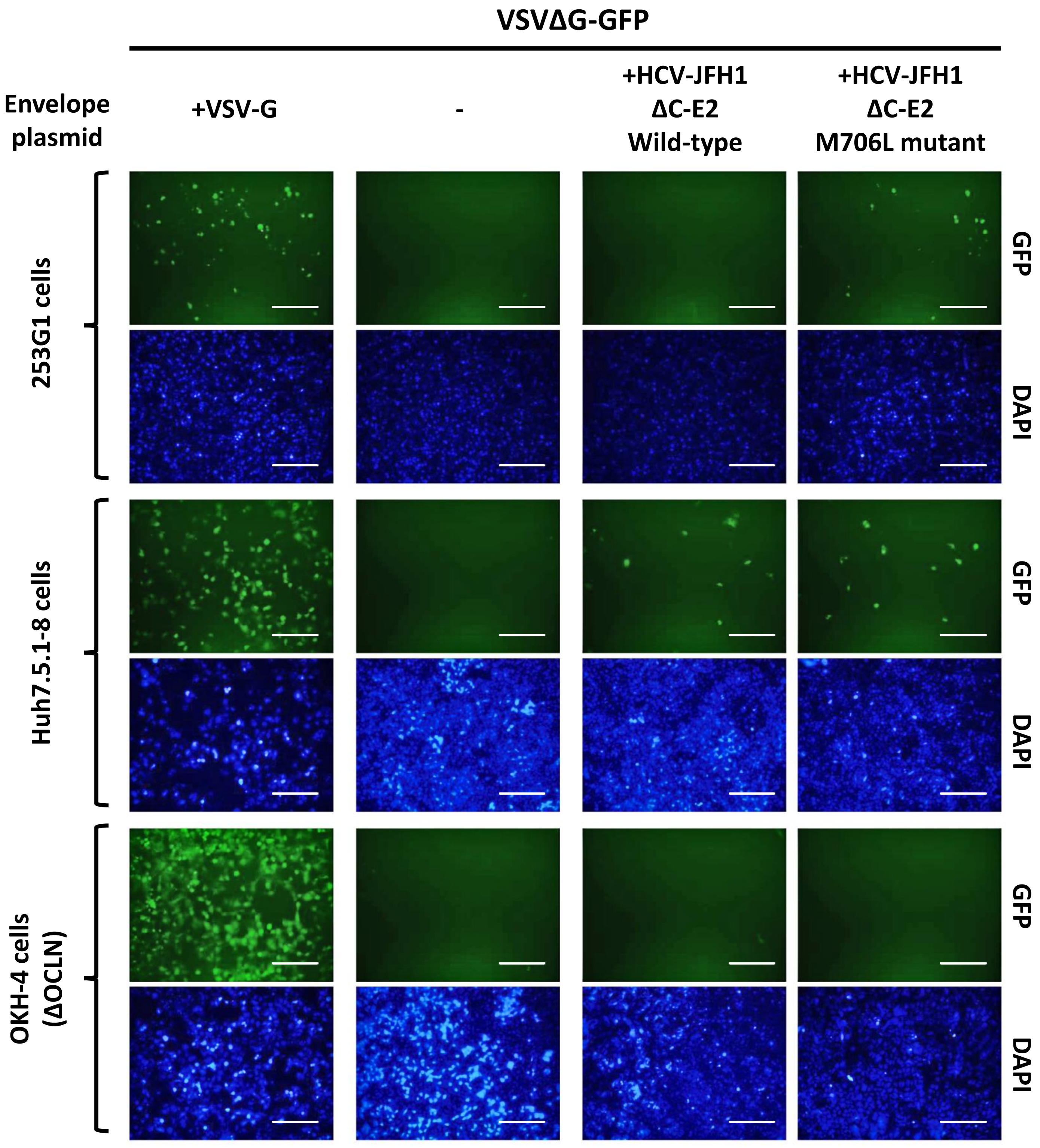

Supplementary Fig. S10

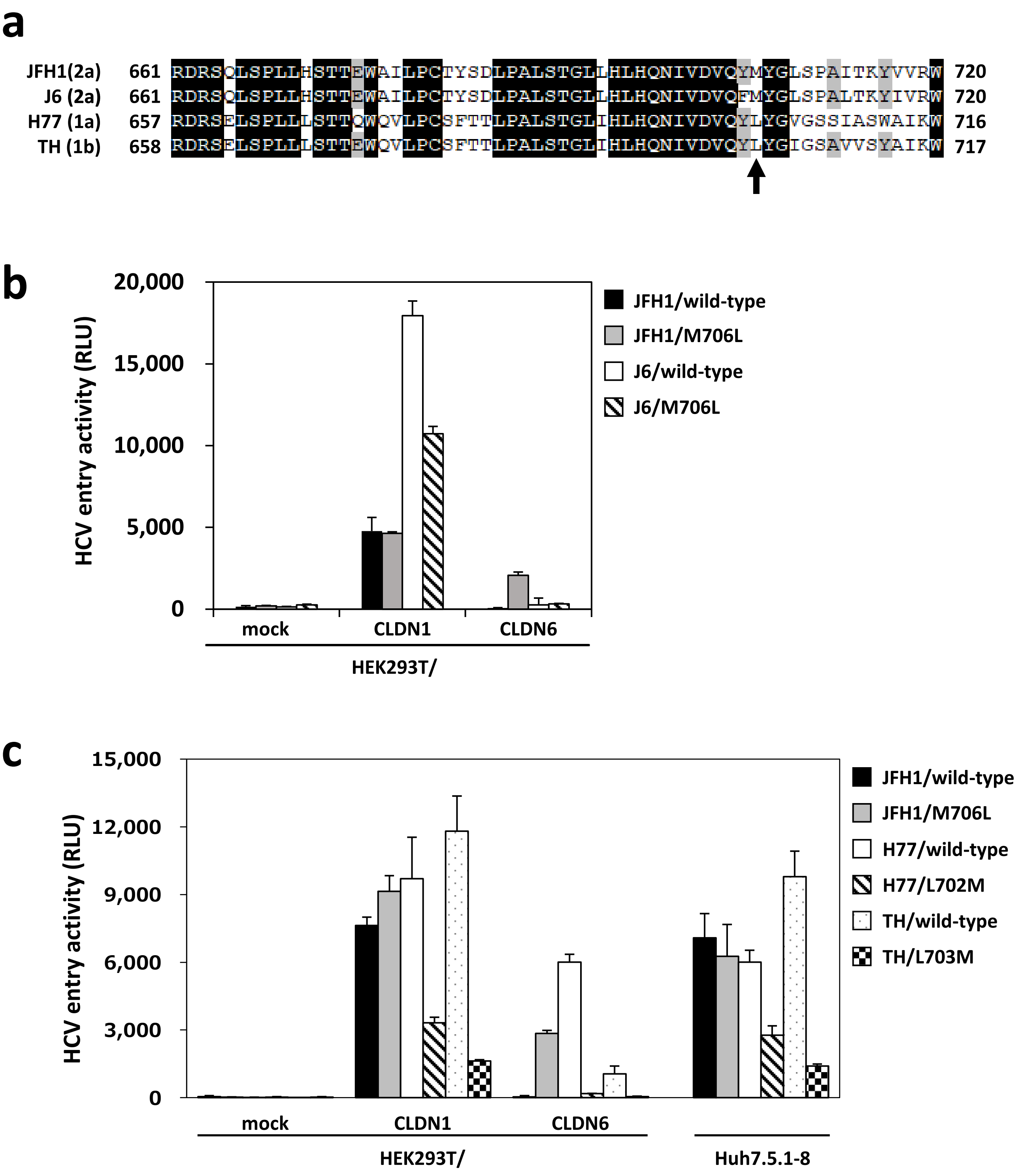

Supplementary Fig. S11

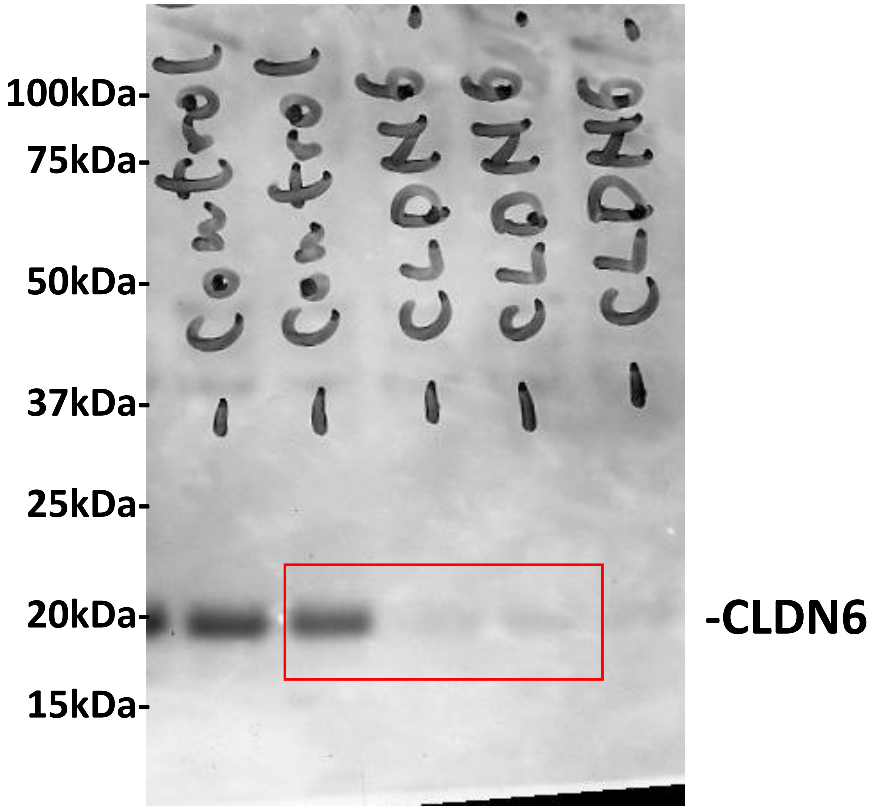

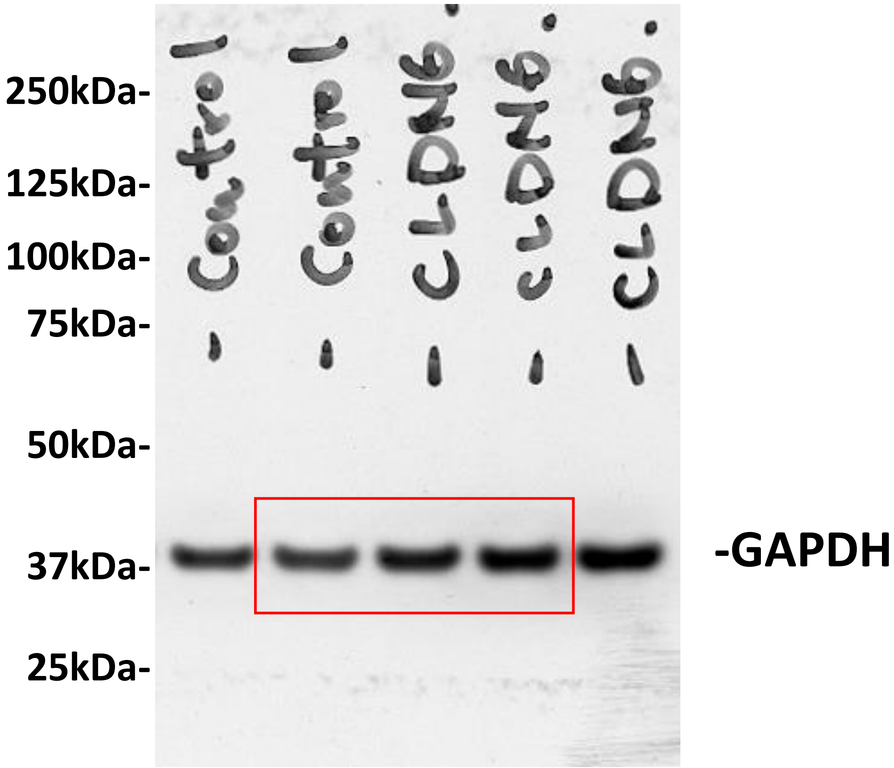

Fig. 5a original blot

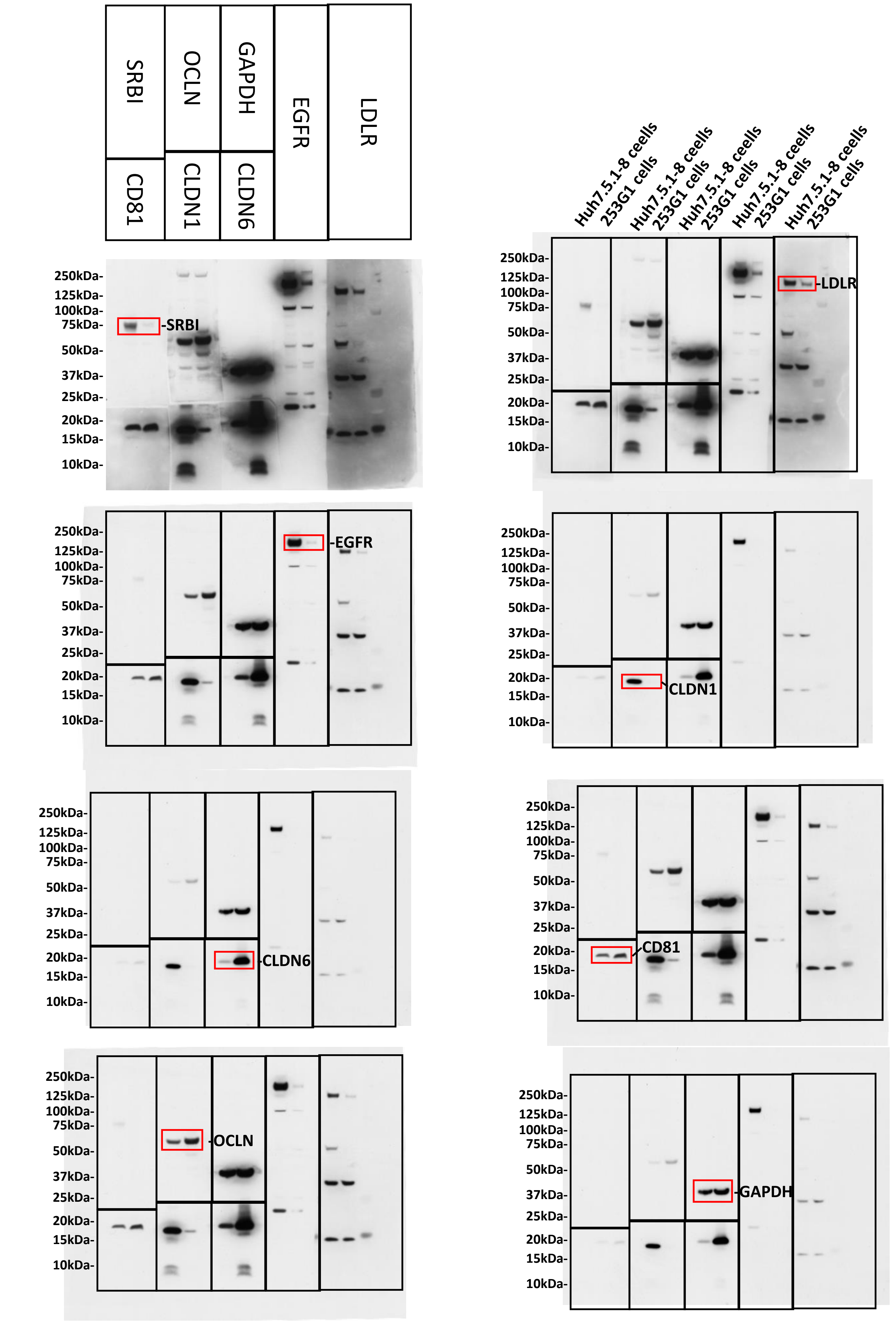

Fig. 6a original blot

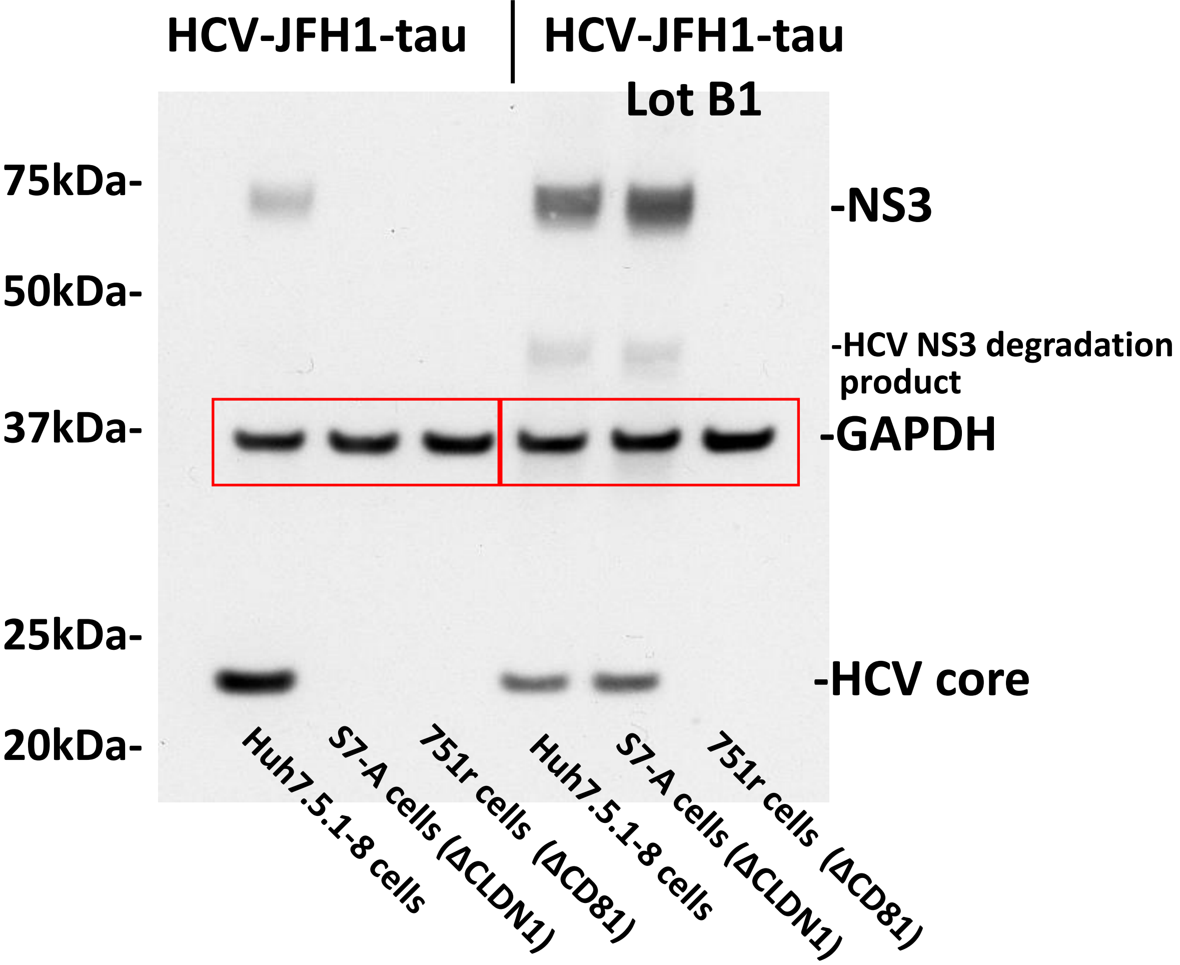

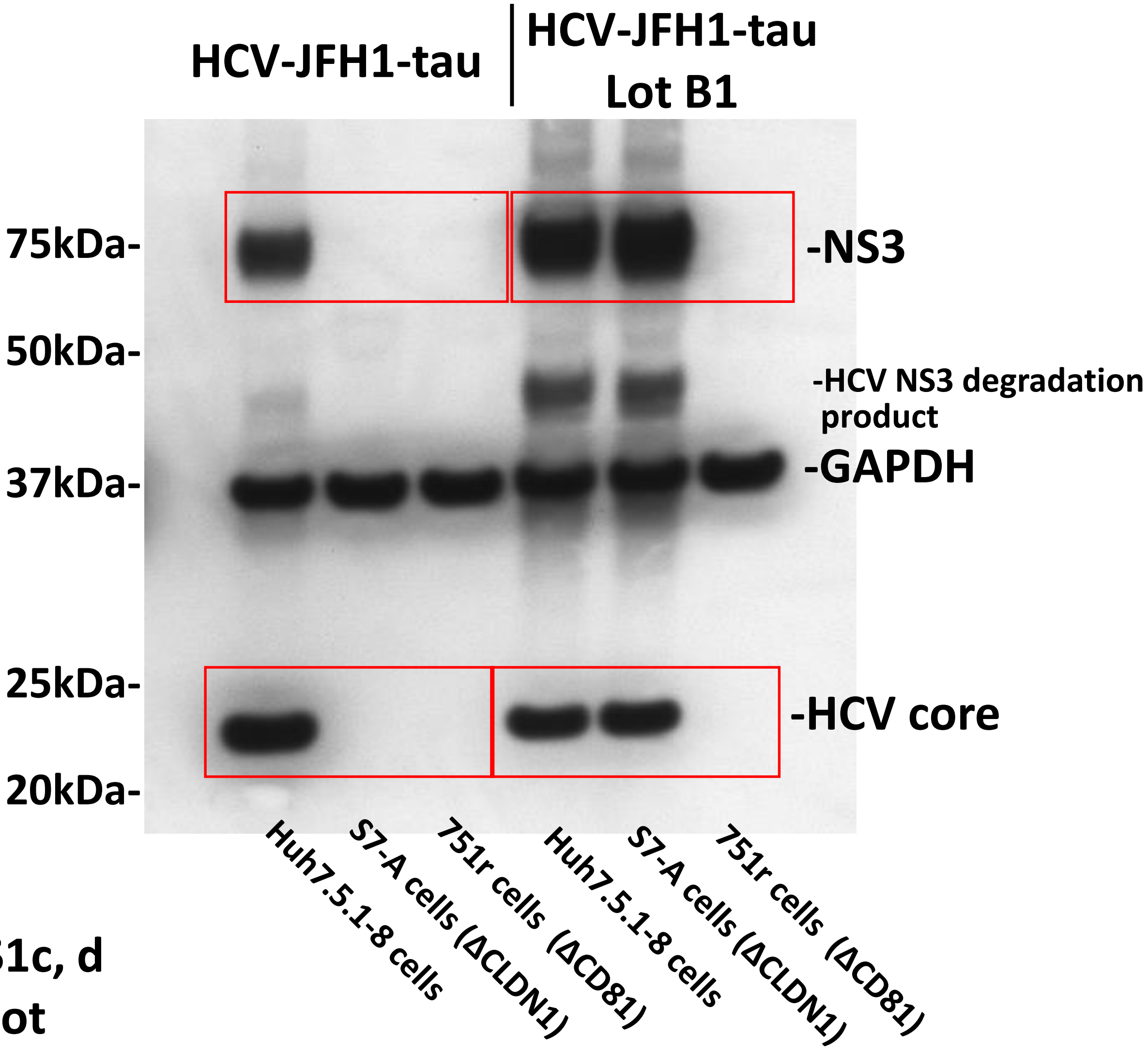

Supl. Fig.S1c, d original blot

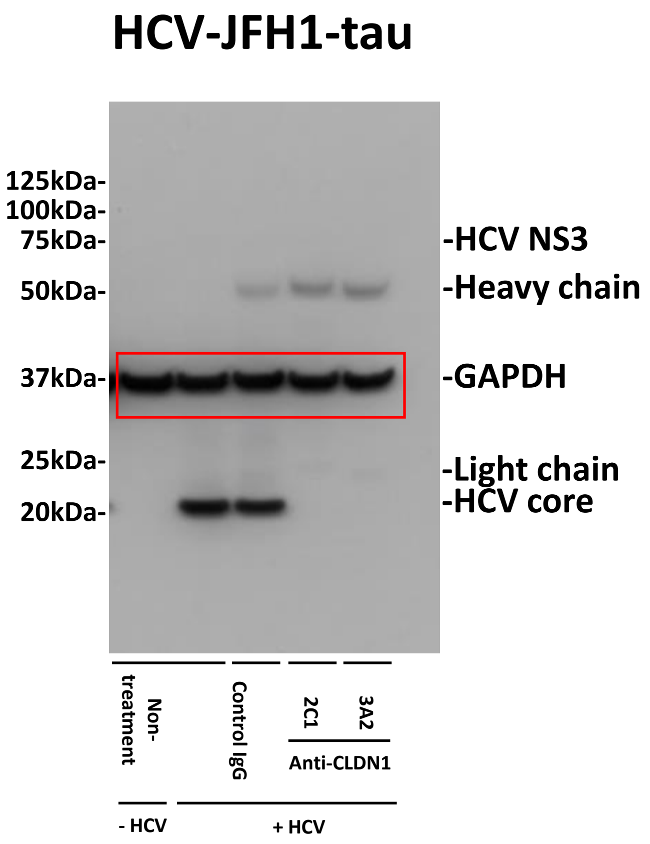

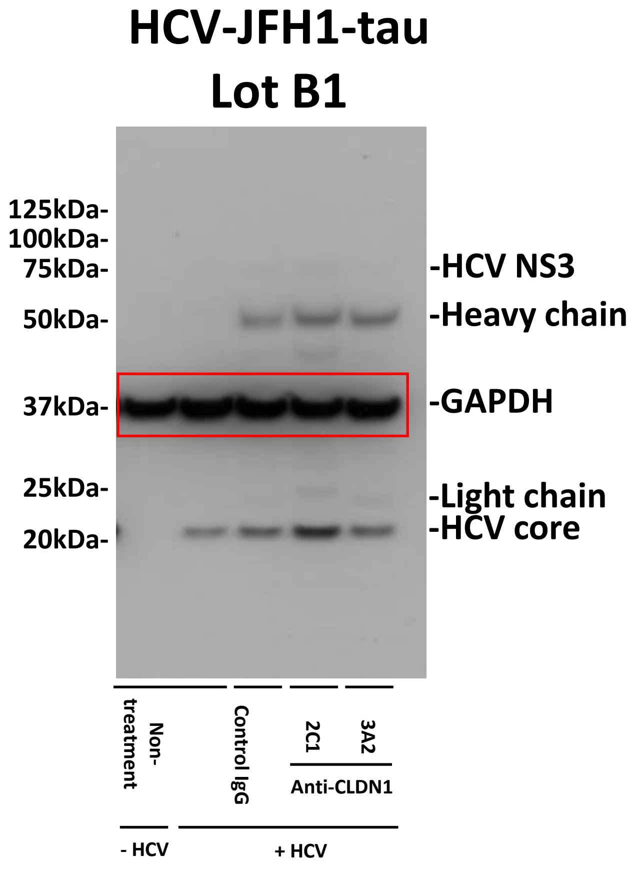

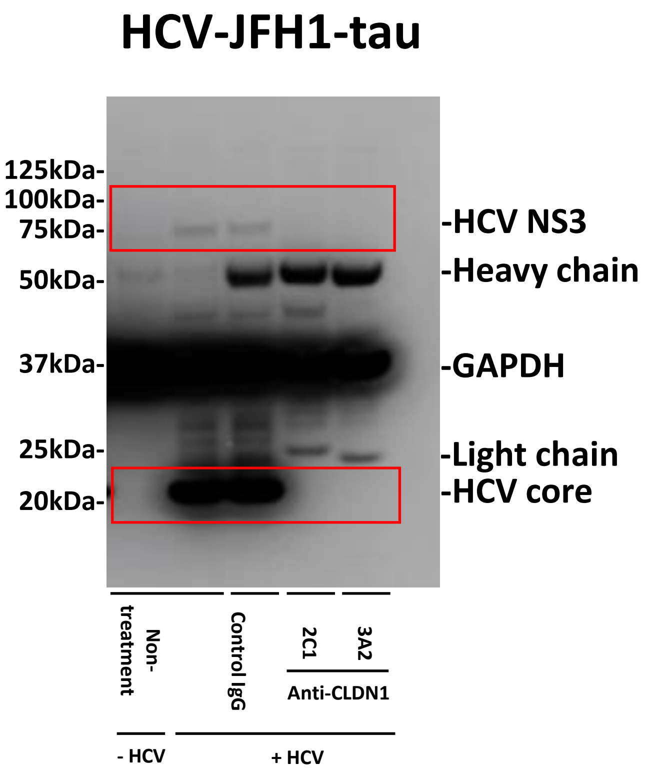

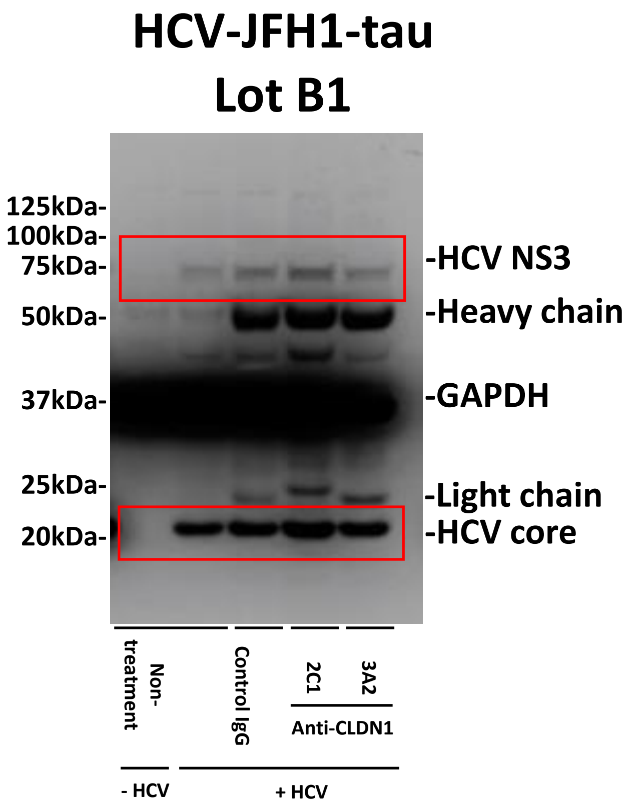

Supl. Fig.S2b original blot

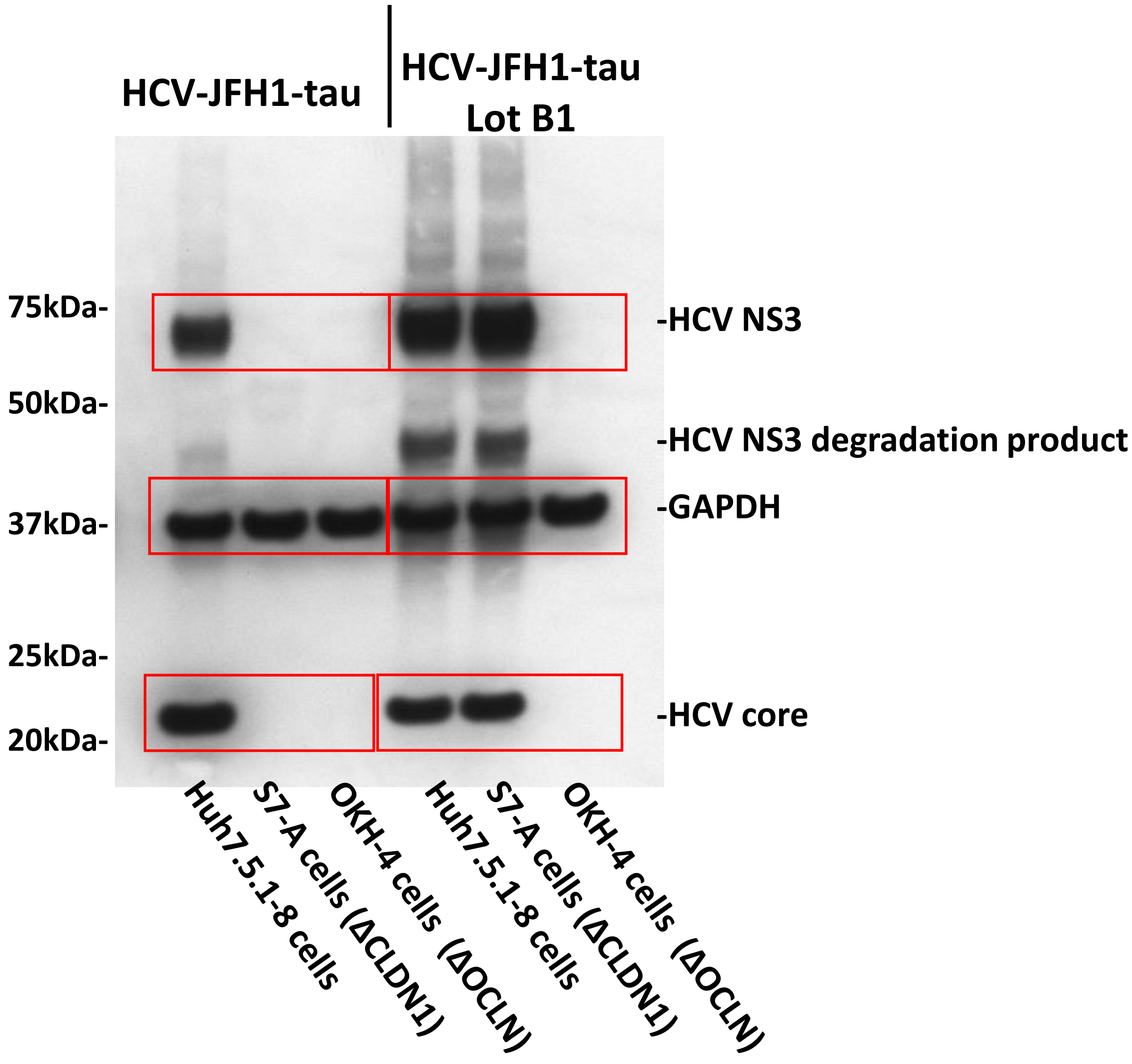

Supl. Fig.S3b original blot

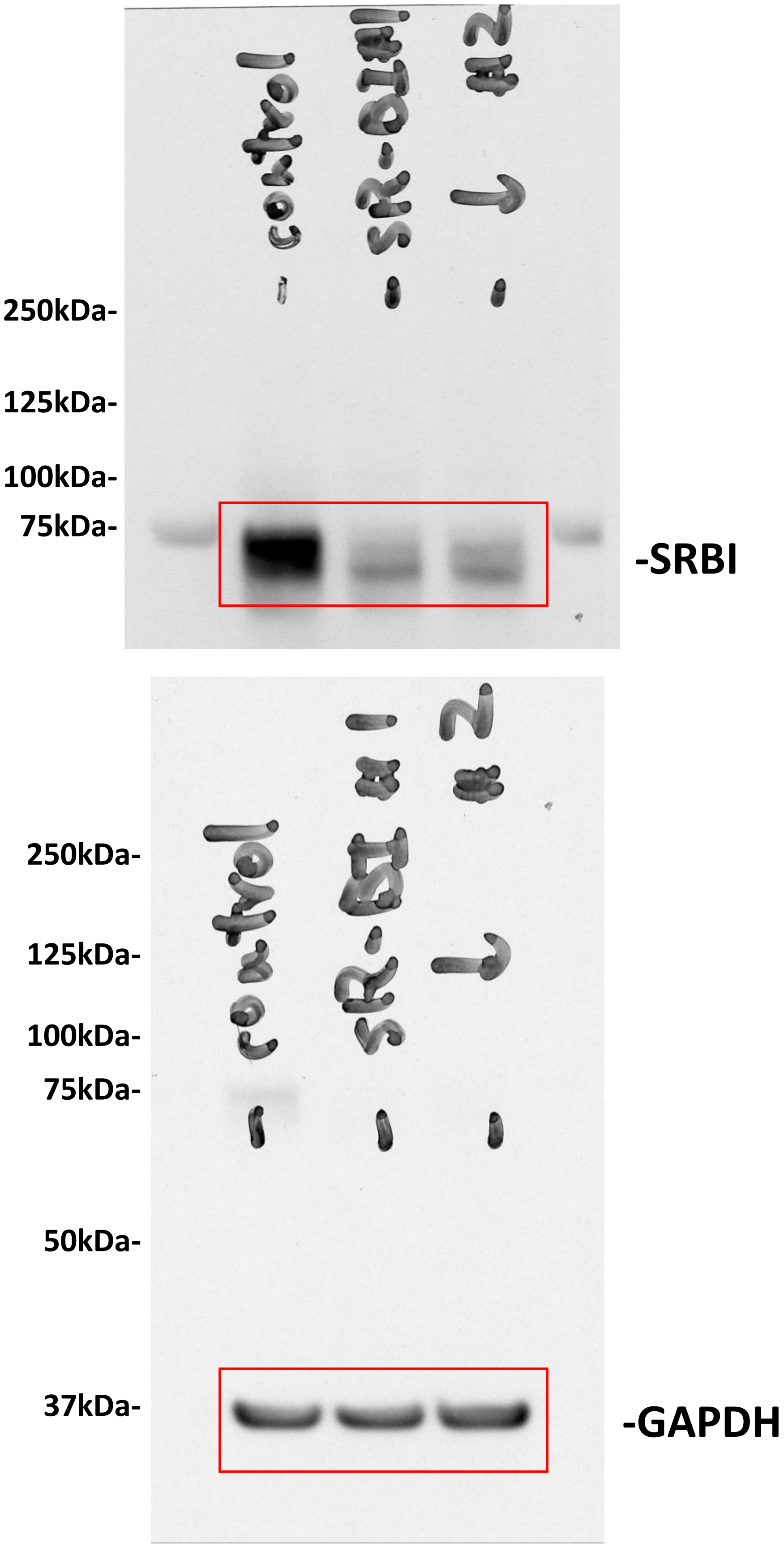

Supl. Fig.S6a original blot

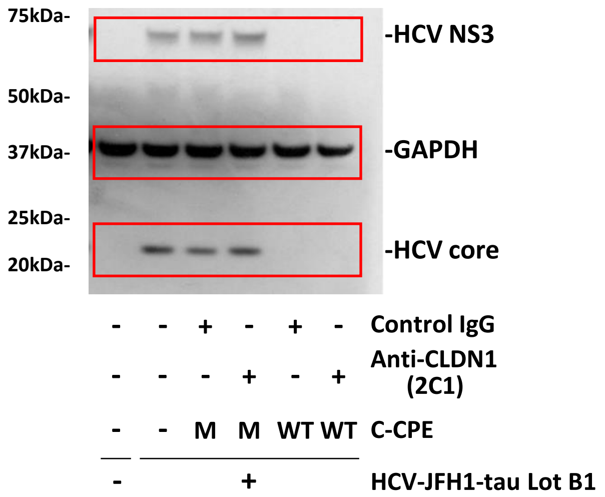

Supl. Fig.S7 original blot
